# A novel homeostatic mechanism tunes PI(4,5)P_2_-dependent signaling at the plasma membrane

**DOI:** 10.1242/jcs.261494

**Published:** 2023-08-29

**Authors:** Rachel C. Wills, Colleen P. Doyle, James P. Zewe, Jonathan Pacheco, Scott D. Hansen, Gerald R. V. Hammond

**Affiliations:** ^1^ Department of Cell Biology, University of Pittsburgh School of Medicine, Pittsburgh, PA 15261, USA; ^2^ Department of Chemistry and Biochemistry, University of Oregon, Eugene, OR 97403, USA

**Keywords:** Phosphoinositide, PtdIns, Signaling, PIPK, Plasma membrane, PI3K, PLC

## Abstract

The lipid molecule phosphatidylinositol (4,5)-bisphosphate [PI(4,5)P_2_] controls all aspects of plasma membrane (PM) function in animal cells, from its selective permeability to the attachment of the cytoskeleton. Although disruption of PI(4,5)P_2_ is associated with a wide range of diseases, it remains unclear how cells sense and maintain PI(4,5)P_2_ levels to support various cell functions. Here, we show that the PIP4K family of enzymes, which synthesize PI(4,5)P_2_ via a minor pathway, also function as sensors of tonic PI(4,5)P_2_ levels. PIP4Ks are recruited to the PM by elevated PI(4,5)P_2_ levels, where they inhibit the major PI(4,5)P_2_-synthesizing PIP5Ks. Perturbation of this simple homeostatic mechanism reveals differential sensitivity of PI(4,5)P_2_-dependent signaling to elevated PI(4,5)P_2_ levels. These findings reveal that a subset of PI(4,5)P_2_-driven functions might drive disease associated with disrupted PI(4,5)P_2_ homeostasis.

## Teaser

**Summary:** The enzyme PIP4K functions as both a sensor and a negative regulator of PI(4,5)P_2_ synthesis by the closely related PIP5K enzymes, tuning the activity of numerous membrane functions.

## INTRODUCTION

The lipid molecule PI(4,5)P_2_ is a master regulator of animal cell plasma membranes (PMs). By recruiting or activating scores of membrane proteins, it controls transport of ions and solutes across the membrane (Dickson and Hille, 2019; Hammond and Burke, 2020), mediates attachment of the underlying cytoskeleton (Saarikangas et al., 2010), regulates the traffic of proteinaceous cargo to and from the membrane (Schink et al., 2015), disseminates extracellular signals (Hammond and Burke, 2020), and facilitates the entry, assembly and egress of bacterial and viral pathogens (Hammond and Burke, 2020; Phan et al., 2019). As a result, synthesis of PI(4,5)P_2_ is essential for life in mammals (Narkis et al., 2007; Volpicelli-Daley et al., 2010). Nonetheless, genetic defects occur in humans that either increase or decrease PI(4,5)P_2_ levels, disrupting cellular physiology in unpredictable ways. These manifest in diseases ranging from cancer (Semenas et al., 2014) to kidney disease (Berquez et al., 2020) and dysentery (Mason et al., 2007). Clearly, there is a central physiological imperative to tightly control PI(4,5)P_2_ levels for harmonious PM function. A detailed homeostatic mechanism that can sense and maintain PI(4,5)P_2_ levels has, however, proven elusive.

**Fig. 1.**
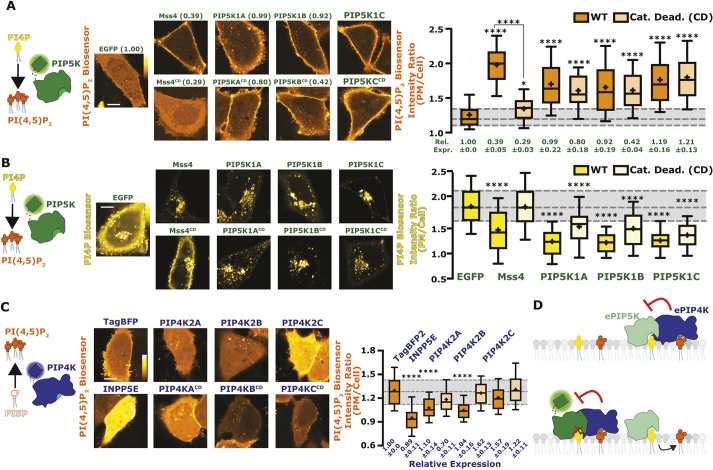
**Reciprocal regulation of PM PI(4,5)P_2_ levels by PIP5K and PIP4K.** (A) PIP5Ks increase PM PI(4,5)P_2_ independently of catalytic activity. Cartoon denotes the catalytic activity of PIP5K. Images show equatorial confocal sections of HeLa cells expressing the low-affinity Tubby_C_^R332H^ PI(4,5)P_2_ sensor (orange), co-transfected with EGFP-tagged catalytically active (WT, wild-type) or dead PIP5K constructs (yeast Mss4 or mammalian A, B or C paralogs), or EGFP alone as control (labels are as for B). Increased PI(4,5)P_2_ is apparent from higher Tubby_C_^R332H^ fluorescence in the PM. Box-and-whisker plots show the mean fluorescence intensity ratio (PM/Cell) of the PI(4,5)P_2_ sensor from >90 cells imaged across at least three independent experiments (boxes displaying median and interquartile range, whiskers representing 10-90% of data and ‘+’ represents mean). Expression of EGFP-tagged proteins relative to the EGFP control is indicated below box-and-whisker plots based on raw fluorescence intensity; data are means±s.e.m. (B) PIP5Ks reciprocally decrease PM PI4P independently of catalytic activity. Cartoon denotes the catalytic activity of PIP5K. Images show equatorial confocal sections of HeLa cells expressing the high affinity P4Mx2 PI4P sensor (yellow), co-transfected with EGFP-tagged catalytically active or dead PIP5K constructs (yeast Mss4 or mammalian A, B or C paralogs), or EGFP alone as control. Decreased PI4P is apparent from loss of P4Mx2 fluorescence at the PM. Box-and-whisker plots are for 88–90 cells imaged across at least three independent experiments as in A. (C) PIP4Ks decrease PM PI(4,5)P_2_ independently of catalytic activity. Cartoon shows the catalytic activity of PIP4K. Images show PI(4,5)P_2_ sensor in HeLa cells as in A, co-transfected with the three different PIP4K paralogs, catalytically dead PIP4K2A or a PI(4,5)P_2_ 5-phosphatase(pptase) (INPP5E). Box-and-whisker plots are for >90 cells imaged across at least three independent experiments as in A. WT forms are is shown in bright orange and catalytically dead in pale orange. Expression of TagBFP2-tagged proteins relative to the TagBFP2 control is indicated below box and whisker plots based on raw fluorescence intensity; data are mean±s.e.m. (D) Proposed inhibition of ePIP5K (endogenous PIP5K) by ePIP4K. With the overexpression of a fluorescently tagged version of PIP5K, regardless of catalytic activity, ePIP4K is sequestered. This relieves endogenous PIP5K from inhibition, increasing PI(4,5)P_2_ levels. Gray shading for box plots in A and C shows median and interquartile range for control. Scale bars: 10 µm. *P<0.05; ****P<0.0001 (full test details provided in Tables S1–S3).

Most prior work in this area has focused on positive regulation of phosphatidylinositol 4-phosphate 5-kinases (PIP5Ks), the major enzymes responsible for PI(4,5)P_2_ synthesis (Fig. 1A). These enzymes add a phosphate to the 5-OH of their substrate, PI4P (Chen et al., 2017; Honda et al., 1999; Jenkins et al., 1994). Such positive regulation can be mediated by the small GTPases Arf6 (Chen et al., 2017; Honda et al., 1999) and Rac (Chao et al., 2010; Halstead et al., 2010) or the PI(4,5)P_2_ metabolite phosphatidic acid (Ishihara et al., 1998). In fact, PIP5Ks cooperatively bind to their product, PI(4,5)P_2_, which creates a positive feedback loop that enhances membrane localization and catalytic output (Hansen et al., 2019). However, we reasoned that maintaining tonic PI(4,5)P_2_ levels in the PM in the presence of abundant PI4P substrate (Hammond et al., 2009, 2014) would demand negative feedback of PIP5Ks. This is especially apparent during lipid re-synthesis after phospholipase C (PLC) activation; PI(4,5)P_2_ levels plateau despite the fact that levels of the precursor lipid PI4P are still rising (Myeong et al., 2021; Tóth et al., 2016; Willars et al., 1998). Potential mechanisms of PI(4,5)P_2_ downregulation include PIP5K autophosphorylation (Itoh et al., 2000), as well as a futile cycle wherein PI(4,5)P_2_ lipids are dephosphorylated back to PI4P by inositol polyphosphate 5-phosphatase (INPP5) enzymes (Myeong et al., 2020), although the specific INPP5 family member(s) responsible for this constitutive activity have not been defined. Finally, PIP5K inhibition by the related phosphatidylinositol 5-phosphate 4-kinases (PIP4Ks), which produce PI(4,5)P_2_ from much less abundant PI5P substrate, has been reported (Wang et al., 2019). However, how this downregulation of PIP5K activity by the PIP4Ks is regulated to maintain PI(4,5)P_2_ homeostasis has not been defined.

A common feature missing from effectors that downregulate PI(4,5)P_2_ synthesis is the identity of sensors that detect changing PI(4,5)P_2_ levels and modulate these effectors appropriately. Without knowledge of such a mechanism, how cells accomplish effective PI(4,5)P_2_ homeostasis and thereby maintain harmonious PM function has been a mystery. In this paper, we demonstrate that the PIP4K family of enzymes act as low-affinity PI(4,5)P_2_ sensors, monitoring tonic PI(4,5)P_2_ levels and constraining PIP5K activity when levels of the lipid rise too high. Modulation of this homeostatic mechanism reveal unprecedented differences in the sensitivity of PI(4,5)P_2_-dependent signaling to resting PI(4,5)P_2_ levels.

## RESULTS

### PIP5Ks are inhibited by PIP4Ks

This study was motivated by some initially perplexing results we obtained when monitoring PM PI(4,5)P_2_ levels with the low-affinity biosensor Tubby_c_^R332H^ (Quinn et al., 2008), together with PI4P levels using the high-affinity biosensor P4Mx2 (Hammond et al., 2014): PI(4,5)P_2_ levels are expected to increase at the expense of PM PI4P levels when overexpressing any of the three paralogs of human PIP5K (PIP5K1A–PIP5K1C) or the single homolog from the budding yeast, *Saccharomyces cerevisiae* (Mss4). Indeed, this was precisely what we observed (Fig. 1A,B, statistics reported in Tables S1 and S2). What perplexed us was that catalytic activity of the human enzymes is dispensable for increased PI(4,5)P_2_ (Fig. 1A) and depleted PM PI4P (Fig. 1B). Catalytic activity is essential for yeast PIP5K, however (Fig. 1A,B). Notably, expression of the catalytically inactive mutants was usually somewhat less strong compared to the wild-type enzymes, yet effects on PI(4,5)P_2_ levels were similar (Fig. 1A).

Conversely, overexpression of PIP4K enzymes, which also make PI(4,5)P_2_ but from PI5P substrate, would be expected to elevate PI(4,5)P_2_ levels slightly. However, we found that PIP4K2A and PIP4K2B actually decreased PM PI(4,5)P_2_ levels, with a ranked order PIP4K2A>PIP4K2B>PIP4K2C (Fig. 1C, statistics in Table S3). For PIP4K2A at least, this occurred even when expressing a catalytically inactive mutant. Again, differences in expression level between paralogs do not explain differences in activity, given that all achieved comparable expression levels as assessed by fluorescence intensity (Fig. 1C). These observations were consistent with a prior report showing that knocking out PIP4K paralogs elevates PI(4,5)P_2_ levels (Wang et al., 2019), because PIP4K enzymes can inhibit PIP5Ks independently of their catalytic activity. We therefore reasoned that saturation of endogenous, inhibitory PIP4K molecules by PIP5K overexpression, regardless of catalytic activity of the PIP5K, would free endogenous, active PIP5K enzyme from negative regulation (Fig. 1D).

**Fig. 2.**
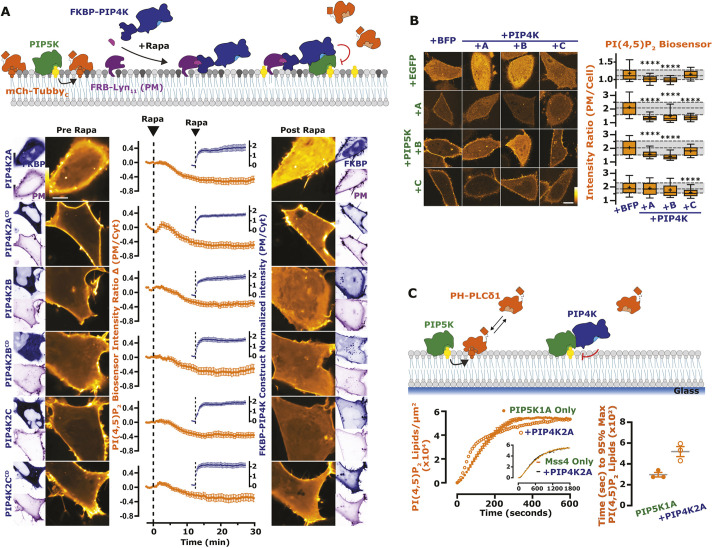
**The activity of PIP5K is blunted by PIP4K.** (A) PIP4K recruitment acutely inhibits PM PI(4,5)P_2_ levels. Cartoon schematics show the chemically induced dimerization (CID) system for FKBP-tagged PIP4K paralogs (A, B, C), which dimerize with the PM-anchored FRB–Lyn_11_ upon the addition of rapamycin (Rapa). HEK293A cells were transfected with FKBP-tagged proteins, the high-affinity PI(4,5)P_2_ indicator Tubby_C_ and FRB–Lyn_11_. During time-lapse confocal microscopy, cells were stimulated with 1 µM Rapa as indicated. Graphs represent mean±s.e.m. change in PI(4,5)P_2_ sensor intensity ratio (PM/Cyt) for 35–60 cells imaged across three independent experiments (orange). Inset graphs show mean±s.e.m. PM recruitment of the FKBP–PIP4K (blue). (B) PIP4Ks antagonize PIP5K-mediated PI(4,5)P_2_ increases. HeLa cells expressing PI(4,5)P_2_ indicator Tubby_C_^R332H^ (orange) were co-transfected with the indicated EGFP- or TagBFP2-tagged constructs. Images show confocal equatorial sections of representative cells. Box-and-whisker plots show the mean fluorescence intensity ratio (PM/Cell) of the PI(4,5)P_2_ sensor from >90 cells imaged across at least three independent experiments (boxes displaying median and interquartile range, whiskers representing 10–90% of data and ‘+’ represents mean). Gray shading shows median and interquartile range of the +BFP controls. (C) PIP4K2A attenuates the kinetics of PI(4,5)P_2_ production driven by PIP5K1A, but not Mss4. Kinetics of PI(4,5)P_2_ production measured on SLBs in the presence of 1 nM PIP5K1A, 20 nM PH-PLCδ1 with or without 50 nM PIP4K2A. Inhibition of PIP5K1A activity is delayed until a threshold density of ∼2% PI(4,5)P_2_ is created to support membrane recruitment of PIP4K2A*.* Inset shows kinetics of reactions executed in the presence of 50 nM Mss4, 20 nM PH-PLCδ1 with or without 50 nM PIP4K2A. Initial membrane composition: 76% DOPC, 20% DOPS, 4% PI(4)P. Right graphs show the quantification of time required for reactions to reach 95% completion (mean±s.e.m.; *n*=3 technical replicates). Scale bars: 10 µm. ****P<0.0001 (full test details provided in Table S4).

To directly test for negative regulation of PIP5K activity by PIP4K in cells, we wanted to assay PI(4,5)P_2_ levels after acute membrane recruitment of normally cytosolic PIP4K paralogs. To this end, we triggered rapid PM recruitment of cytosolic, FKBP-tagged PIP4K by chemically induced dimerization (CID) with a membrane-targeted FRB domain, using rapamycin (Varnai et al., 2006). As shown in Fig. 2A, all three paralogs of PIP4K induce a steady decline in PM PI(4,5)P_2_ levels within minutes of PM recruitment. Catalytically inactive mutants of all three paralogs produce identical responses (Fig. 2A).

We also reasoned that co-expression of PIP4K paralogs with PIP5K might attenuate the elevated PI(4,5)P_2_ levels induced by the latter. Broadly speaking, this was true, but with some curious paralog selectivity (Fig. 2B, statistics reported in Table S4). PIP4K2A and PIP4K2B both attenuated PI(4,5)P_2_ elevated by PIP5K1A and PIP5K1B, but not (or much less so) for that elevated by PIP5K1C; PIP4K2C, on the other hand, attenuated PIP5K1A and was the only paralog to significantly attenuate the effect of PIP5K1C, yet it did not attenuate PIP5K1B at all.

To more directly examine inhibition of PIP5K by PIP4K, we tested activity of purified PIP5K1A on PI4P-containing supported lipid bilayers (SLBs). Addition of PIP4K2A exhibited delayed inhibition of PIP5K1A activity (Fig. 2C): Once PI(4,5)P_2_ reached ∼28,000 lipids/µm^2^ (∼2 mol %), PIP5K-dependent lipid phosphorylation slowed down, which doubled the reaction completion time (Fig. 2C, right). In contrast, we observed no PIP4K-dependent inhibition of Mss4 (Fig. 2C, inset). These data recapitulate the prior finding that PIP4K only inhibited purified PIP5K in the presence of bilayer-presented substrate (Wang et al., 2019). We therefore hypothesized that inhibition of PIP5K by PIP4K requires recruitment of the latter enzyme to the PM by PI(4,5)P_2_ itself.

### PIP4Ks are low-affinity sensors of PM PI(4,5)P_2_

**Fig. 3.**
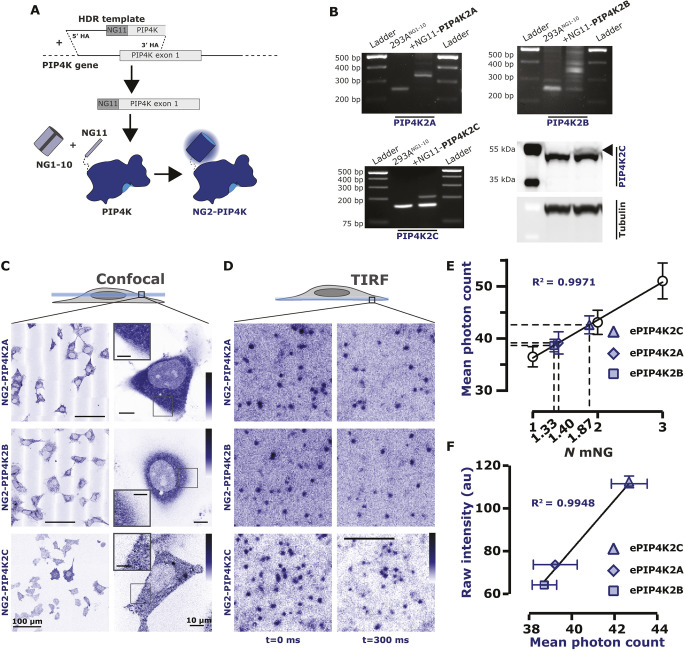
**Endogenously tagged PIP4Ks are associated with the PM.** (A) Endogenous tagging of PIP4K. Brief cartoon schematic showing the mechanism of endogenous tagging employed for PIP4K with NeonGreen2 (NG2). The resulting cell lines were termed NG2–PIP4K. This strategy was used for all three paralogs of PIP4K. (B) For PIP4K2A and PIP4K2B paralogs, cells were genotyped with paralog-specific forward and reverse primer yielding an edited product of ∼350 bp. For PIP4K2C, cells were genotyped with a NG2-specific forward primer and a PIP4K2C paralog specific reverse primer yielding an edited product of ∼200 bp. Details of primers are provided in Table S10. NG2–PIP4K2C cells were also probed with a PIP4K2C specific antibody showing the expected ∼3 kDa shift in molecular mass (arrowhead). (C) Confocal-based characterization of NG2–PIP4K. Confocal images displaying the NG2–PIP4K (blue) in cells, which localized mainly to the cytosol. In the case of NG2–PIP4K2C, a slight association of the enzyme to the PM can be seen in the magnifications. Scale bars: 100 µm (overview); 10 µm (magnification); 5 µm (inset magnification). (D) TIRF-based characterization of NG2–PIP4K. When imaged live by TIRFM, dynamic, diffraction limited spots are observed on the membrane (compare differential localization at 0 and+300 ms). Scale bar: 2.5 µm. Images in C and D are representative of at least three experimental repeats. (E) Endogenous PIP4Ks exists as heterogeneous populations. All three PIP4K paralogs have an intensity consistent with a mixed population of one or two mNG molecules when calibrated against single, dimeric or trimeric mNG molecules fused to a PI(4,5)P_2_-binding domain. This correlates to the mean photon count of heterogeneously tagged cell populations (one or two alleles tagged with NG2^11^). Data are grand mean photon counts ±95% c.i. for data acquired from 22–43 cells. The linear regression and resulting R^2^ against the grand mean values are also plotted. (F) Endogenous expression levels vary between PIP4K paralogs. The raw fluorescence intensity of NG2–PIP4K2A, NG2–PIP4K2B or NG2–PIP4K2C in each tagged cell line was measured and plotted against the mean photon counts from E. Plotted points show the raw fluorescence intensity of each NG2–PIP4K paralog from 90 cells imaged across three independent experiments (points display the mean±s.e.m.). There is a positive correlation between the expression level of each paralog and its likelihood to be visualized as a monomeric versus a dimeric protein (the linear regression and resulting R^2^ against mean values are plotted). PIP4K2A and PIP4K2B can exist as either homodimers or more likely heterodimers; whereas PIP4K2C more likely homodimerizes. au, arbitrary units.

To probe the interaction of endogenous PIP4Ks with PM PI(4,5)P_2_, we used a split fluorescent protein genome editing approach (Feng et al., 2017) to add a NeonGreen2 (NG2) tag to each of the three PIP4K paralogs (Fig. 3A). Successful integration of the split NG2 tag was evident at the genomic level (Fig. 3B); a minor shift in protein size was also observed at the protein level after addition of the neonGreen^11^ tag to PIP4K2C (Fig. 3B). As expected, endogenous PIP4Ks have a mainly cytosolic distribution as viewed by confocal microscopy, with a slight enrichment at the cell periphery (Fig. 3C), which is consistent with results from the OpenCell project (Cho et al., 2022).

Analysis of the ventral PM by total internal reflection fluorescence microscopy (TIRFM) revealed individual, diffraction-limited and uniform intensity puncta that were dynamically associated with the membrane (Fig. 3D). We compared the intensity of these puncta with a PI(4,5)P_2_ biosensor tagged with single, double or triple mNeonGreen copies expressed at single-molecule levels. This revealed that the NG2–PIP4K2C puncta contained an average of 1.87 NG2 molecules, whereas NG2–PIP4K2A puncta contained 1.40 and NG2–PIP4K2B contained 1.33 NG2 molecules. This is consistent with dimeric PIP4K complexes (Rao et al., 1998) displaying lower fluorescence due to heterodimerization between NG2–PIP4Ks and unlabeled endogenous PIP4Ks (Fig. 3E). Analysis of the average fluorescence intensity of confocal sections of the edited cells recapitulates the ranked expression order of the PIP4Ks in HEK293 cells seen in proteomic studies (Cho et al., 2022; Geiger et al., 2012), with PIP4K2C>>PIP4K2A>PIP4K2B (Fig. 3F). Satisfyingly, the total intensity of the cells scales linearly with the photon count of single NG2-containing complexes resolved as puncta (Fig. 3F); this is expected, given that PIP4K paralogs exist as a series of randomly associated homo- and hetero-dimers of the three paralogs (Wang et al., 2010). Therefore, NG2-PIP4K2C dimers are expected to be more frequent given that there is more total PIP4K2C expression in HEK293 and thus a higher probability of homodimerization between molecules of this paralog.

**Fig. 4.**
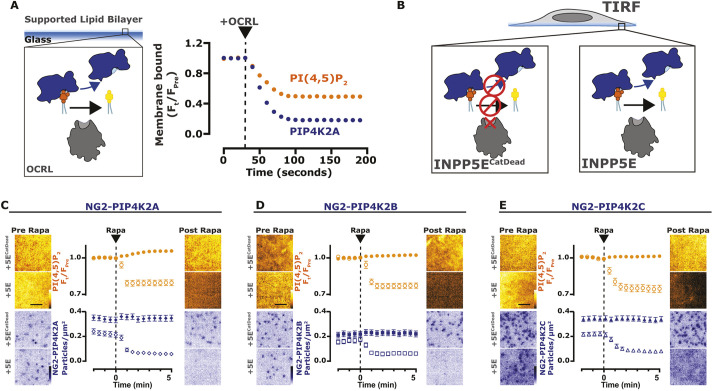
**PI(4,5)P_2_ is necessary for the PM localization of PIP4K.** (A) Depletion of PI(4,5)P_2_ causes PIP4K2A to dissociate from SLBs. Imaging chambers containing 50 nM PIP4K2A and 20 nM PH-PLCδ1 at equilibrium with SLBs composed of 96% DOPC and 4% PI(4,5)P_2_ were visualized by TIRF microscopy. At 30 s, 100 nM OCRL was added to catalyze the dephosphorylation of PI(4,5)P_2_ and membrane dissociation of PIP4K2A and PH-PLCδ1. (B) Depletion of PI(4,5)P_2_ causes NG2–PIP4K2C to dissociate from the membrane. Cartoons show the CID system, in TIRFM, for FKBP-tagged INPP5E (catalytically active or dead) dimerizing with the PM-anchored Lyn_11_–FRB. (C) Depletion of PI(4,5)P_2_ causes NG2–PIP4K2A to dissociate from the membrane. NG2–PIP4K2A (blue) cells were transfected with FKBP-tagged proteins, the high affinity PI(4,5)P_2_ indicator Tubby_C_ (orange) and Lyn_11_-FRB. During time-lapse TIRF microscopy, cells were stimulated with 1 µM Rapa, as indicated. Tubby_C_ traces represent mean±s.e.m. change in fluorescence intensity (*F*_t_/*F*_pre_) The NG2–PIP4K2A traces represent the mean±s.e.m. change in puncta per µm^2^. A total of 29–32 cells were imaged across three independent experiments. (D) Depletion of PI(4,5)P_2_ causes NG2–PIP4K2B to dissociate from the membrane. As in C, NG2–PIP4K2B (blue) cells were transfected with FKBP-tagged proteins, Tubby_C_ (orange) and Lyn_11_–FRB; cells were stimulated with 1 µM Rapa, as indicated. Tubby_C_ traces represent mean±s.e.m. change in fluorescence intensity (*F*_t_/*F*_pre_). The NG2–PIP4K2B traces represent the mean±s.e.m. change in puncta per µm^2^. A total of >32 cells were imaged across three independent experiments. (E) Depletion of PI(4,5)P_2_ causes NG2–PIP4K2C to dissociate from the membrane. As in C, NG2–PIP4K2C (blue) cells were transfected with FKBP-tagged proteins, Tubby_C_ (orange) and Lyn_11_–FRB; cells were stimulated with 1 µM Rapa, as indicated. Tubby_C_ traces represent mean±s.e.m. change in fluorescence intensity (*F*_t_/*F*_pre_). The NG2–PIP4K2C traces represent the mean±s.e.m. change in puncta per µm^2^. A total of >38 cells were imaged across three independent experiments. Scale bars: 2.5 µm.

Given the dynamic interaction of all three PIP4K paralogs with the PM, we next asked the question: does this interaction depend on PI(4,5)P_2_? On supported lipid bilayers, purified PIP4K2A was released from the membrane upon depletion of PI(4,5)P_2_ by the addition of the 5-OH phosphatase, OCRL (Fig. 4A), mirroring the kinetics of the PH-PLCδ1 lipid biosensor. To determine whether this also holds true with native proteins in living cells, we employed CID to recruit the INPP5E 5-OH phosphatase to rapidly deplete PM PI(4,5)P_2_ (Varnai et al., 2006). As shown in Fig. 4C–E, PI(4,5)P_2_ depletion was evident from the rapid loss of the high-affinity biosensor Tubby_c_ (Quinn et al., 2008). This depletion was accompanied by loss of PM-localized molecules of all three NG2–PIP4K paralogs, PIP4K2A (Fig. 4C), PIP4K2B (Fig. 4D) and PIP4K2C (Fig. 4E) when viewed by TIRFM. Therefore, PI(4,5)P_2_ is necessary to drive PIP4K association with the membrane.

**Fig. 5.**
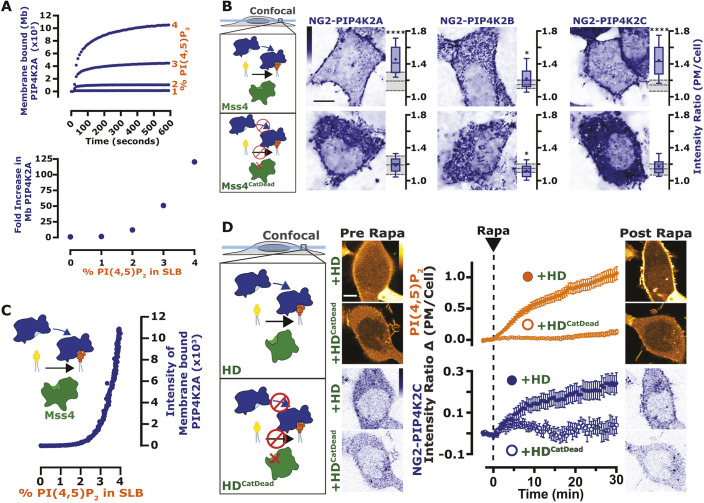
**PIP4K is a low-affinity PI(4,5)P_2_-sensing protein.** (A) Purified PIP4K2A localizes to PI(4,5)P_2_ in a concentration-dependent manner. Membrane absorption and equilibration kinetics of 50 nM Alexa488–PIP4K2A measured by TIRFM on SLBs containing 1–4% PI(4,5)P_2_. PIP4K2A membrane binding exhibited non-linearity with respect to the PI(4,5)P_2_ lipid density. Quantification of the fold increase in membrane-bound PIP4K2A relative to the equilibrium fluorescence intensity of PIP4K2A on a membrane containing 0% PI(4,5)P_2_ is shown in the lower panel. Results representative of at least three repeats. (B) Chronic enrichment of PI(4,5)P_2_ causes NG2–PIP4K paralogs to associate with the membrane. Cartoons show the expression of catalytically active or dead Mss4. Images show equatorial confocal sections of representative NG2–PIP4K cells transfected with Mss4. Box-and-whisker plots show the mean fluorescence intensity ratio (PM/Cell) of the indicated NG2–PIP4K paralog from 90 cells imaged across at least three independent experiments (boxes display median and interquartile range, whiskers represent 10–90% of data and ‘+’ represents mean. Gray shading show medians and interquartile range of TagBFP2-only controls. *P<0.05; ****P<0.0001 (full test details provided in Table S5). (C) Enrichment of PI(4,5)P_2_ causes dynamic membrane recruitment of purified PIP4K2A. In SLBs, membrane recruitment of 50 nM PIP4K2A monitored during Mss4 catalyzed phosphorylation of PI(4)P. Membranes containing 4% PI(4)P were converted into having PI(4,5)P_2_ using 10 nM Mss4. Results representative of at least three repeats. (D) Acute enrichment of PI(4,5)P_2_ causes PIP4K2C to increase association with the membrane. Cartoons show the CID system, in confocal, for the interaction of catalytically active or dead FKBP-tagged homo-dimeric PIP5K1C kinase (HD) with the PM-anchored Lyn_11_–FRB. NG2–PIP4K2C (blue) cells were transfected with FKBP-tagged proteins, the low-affinity PI(4,5)P_2_ indicator Tubby_C_^R332H^ (orange) and Lyn_11_–FRB. During time-lapse confocal microscopy, cells were stimulated with 1 µM Rapa, as indicated. Traces represent mean±s.e.m. change in fluorescence intensity (change in PM/Cell ratio from pre-stimulation levels) of 48–52 cells imaged across at least three independent experiments. Scale bars: 10 µm.

Despite this clear PI(4,5)P_2_ binding, a relatively small fraction of PIP4K2C is present on the PM at steady state (see confocal images in Fig. 3C). Given that there are many orders of magnitude more PI(4,5)P_2_ molecules in the PM than PIP4K in the cell (Wills and Hammond, 2022), these observations suggest that PIP4Ks bind the lipid with low affinity. Indeed, PIP4K2A binding to supported lipid bilayers was barely evident at 1% PI(4,5)P_2_, but detectable at 2% and rose sharply at 3 and 4% (Fig. 5A). This is suggestive of a highly co-operative binding mode, as might be expected from a dimeric protein. Notably, binding was not saturated at these low lipid mole fractions, which are thought to be physiological (Wills and Hammond, 2022). We therefore reasoned that elevating PM PI(4,5)P_2_ levels might actually increase endogenous PIP4K association with the PM. To this end, we employed overexpression of Mss4, given that this enzyme does not bind to PIP4Ks (Fig. 2C) and enhances PI(4,5)P_2_ in a manner that depends on catalytic activity (Fig. 1A). Overexpression of Mss4 indeed enhanced membrane binding of all three PIP4K paralogs in a manner dependent on catalytic activity (Fig. 5B, statistics reported in Table S5). On PI4P-containing supported lipid bilayers, the addition of active Mss4 induced PIP4K2A binding to the lipid bilayer, again with evidence of co-operativity and a threshold of ∼2% PI(4,5)P_2_ (Fig. 5C).

We next tested for rapid binding to acutely increasing PI(4,5)P_2_ levels in living cells, using CID of a homodimeric mutant PIP5K domain (PIP5K-HD), which can only dimerize with itself and not endogenous PIP5K paralogs (Hu et al., 2015). This domain also lacks two basic residues that are crucial for membrane binding (Arioka et al., 2003), and only elevates PM PI(4,5)P_2_ when it retains catalytic activity (Fig. 5D), unlike the full-length protein (Fig. 1A). Recruitment of the active mutant PIP5K domain acutely elevated NG2–PIP4K2C membrane association with identical kinetics to the Tubby_c_^R332H^ PI(4,5)P_2_ reporter, whereas the catalytically inactive mutant was without effect (Fig. 5D).

**Fig. 6.**
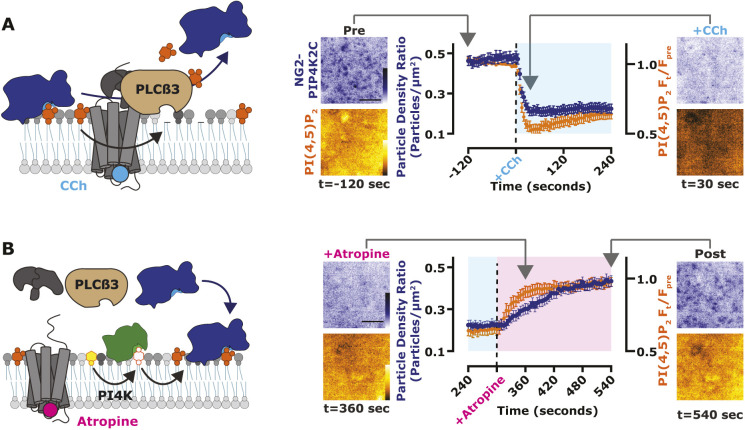
**PIP4K binds to the PM at elevated PI(4,5)P_2_ levels.** (A) PM localization of PIP4K2C follows depletion of PI(4,5)P_2_. Cartoons show PLCβ3-mediated loss of PI(4,5)P_2_ and NG2–PIP4K2C. NG2–PIP4K2C (blue) cells were transfected with the muscarinic acetylcholine receptor M3 and the high-affinity PI(4,5)P_2_ indicator Tubby_C_ (orange). During time-lapse TIRF microscopy, cells were stimulated with 100 µM of the M3 agonist, carbachol (CCh), as indicated. Tubby_C_ traces represent mean±s.e.m. change in fluorescence intensity (*F*_t_/*F*_pre_). The NG2–PIP4K traces represent the mean±s.e.m. change in puncta per µm^2^. A total of 40 cells were imaged across at least three independent experiments. (B) PM localization of PIP4K2C follows resynthesis of PI(4,5)P_2_. Cartoons show the reappearance of PI(4,5)P_2_ and NG2–PIP4K2C after addition of the M3 receptor antagonist, atropine. The data are from the later phase of the experiment depicted in A. During time-lapse TIRFM, cells were stimulated with 5 µM atropine, as indicated. Scale bars: 2.5 µm.

These data indicate that PIP4K2C binds PM PI(4,5)P_2_ with relatively low affinity. As an additional test of this in live cells, we assessed the kinetics of PM binding during PI(4,5)P_2_ re-synthesis after strong PLC activation. Stimulation of overexpressed PLC-coupled muscarinic M3 receptors induced rapid depletion of both NG2–PIP4K2C and PI(4,5)P_2_ (measured with Tubby_c_, Fig. 6A). Subsequent induction of PI(4,5)P_2_ re-synthesis with the muscarinic antagonist atropine revealed much slower rebinding of NG2–PIP4K2C to the PM compared to the Tubby_c_ PI(4,5)P_2_ biosensor; PIP4K2C takes more than twice as long (Fig. 6B).

Collectively, these data demonstrate that PIP4Ks are low-affinity PI(4,5)P_2_ effectors, poised to sense both decreases and crucially, elevations in PI(4,5)P_2_ levels in the PM. Combined with the previously identified inhibition of PIP5K activity by PIP4K (Wang et al., 2019 and Fig. 2), this suggests a mechanism where PIP4K can act as both receptor and control center for PI(4,5)P_2_ homeostasis, with PIP5K as the effector: when PI(4,5)P_2_ levels rise due to PIP5K activity, PIP4K is recruited to the PM, where it can directly bind and inhibit PIP5K. However, such a mechanism suggests a direct interaction of PIP5K and PIP4K. It is to this question that we turn our attention next.

### PIP4K directly interacts with PIP5K

**Fig. 7.**
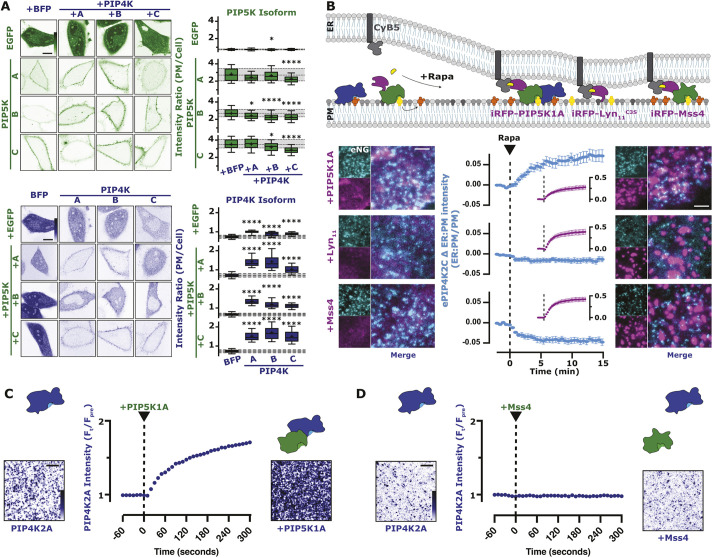
**PIP4K directly interacts with mammalian PIP5K.** (A) PIP5K expression increases PIP4K PM localization. The same experimental data set from Fig. 1E is used here. HeLa cells expressing PIP5K (green) or PIP4K (blue) were co-transfected with the indicated EGFP- or TagBFP2-tagged paralog constructs. Images show equatorial sections in confocal of representative cells. Scale bars: 10 µm. For box-and-whisker plots, boxes display median and interquartile range, whiskers representing 10–90% of the data and ‘+’ representing the mean, of 90 cells imaged across at least three independent experiments. Gray shading shows median and interquartile range of the BFP controls. *P<0.05; ****P<0.0001 (full test details are provided in Table S6). (B) PIP4K2C interacts with PIP5K1A. Cartoon schematics show the CID system for the generation of ER–PM contact sites between ER-anchored FKBP–CyB5 and PM-anchored FRB-tagged constructs. NG2–PIP4K2C (cyan) cells were transfected with FKBP–CyB5, mCherry–MAPPER and the indicated FRB-tagged construct (magenta). During time-lapse TIRFM, cells were stimulated with 1 µM Rapa. TIRF images are representative and color-coded to represent fluorescence intensity, as indicated. Scale bars: 2.5 µm. NG2–PIP4K2C traces represent mean±s.e.m. fluorescence intensities (ER:PM/PM) of 32–39 cells imaged across a minimum of three independent experiments. (C) Dynamic PIP5K1A-dependent membrane recruitment of PIP4K2A to SLBs. TIRFM images show the membrane localization of PIP4K2A in the absence and presence of PIP5K1A. In the absence of PIP5K, 50 nM PIP4K2A displays a low level of membrane recruitment. The addition of 10 nM PIP5K1A, stimulates an immediate and steady increase in PIP4K2A membrane localization. Membrane composition: 2% PI(4,5)P_2_, 98% DOPC. TIRF images are representative and color-coded to represent fluorescence intensity, as indicated. (D) Membrane binding of PIP4K2A is insensitive to yeast Mss4 membrane localization. TIRFM images show the membrane localization of PIP4K2A in the absence and presence of Mss4. Following membrane equilibration of 50 nM PIP4K2A, 10 nM Mss4 was added to the imaging chamber. No appreciable change in PIP4K2A localization was observed during membrane absorption of Mss4. Membrane composition: 2% PI(4,5)P_2_ and 98% DOPC. TIRF images are representative and color-coded to represent fluorescence intensity, as indicated. Results in C and D are representative of at least three repeats. Scale bars: 5 µm.

Previous evidence in the literature points to direct interactions between PIP5Ks and PIP4Ks. Overexpressed PIP4K2A is able to co-immunoprecipitate different PIP5K paralogs (Hinchliffe et al., 2002), and epitope-tagged PIP5K1A is able to pulldown PIP4K2A when expressed at close to endogenous levels (Wang et al., 2019). When co-expressing EGFP-tagged PIP5Ks and TagBFP2-tagged PIP4K2s, we found that the PM binding of PIP5K paralogs is largely unaffected by PIP4K overexpression (Fig. 7A, upper panel and Table S6), whereas all three paralogs of PIP4K are strongly recruited to the PM by co-expression of any PIP5K (Fig. 7A, lower panel and Table S7), as previously observed for PIP4K2A (Hinchliffe et al., 2002).

Although these data are consistent with a direct interaction between PIP4Ks and PIP5Ks, another possibility exists: the PIP5K dependent increase in PI(4,5)P_2_ (Fig. 1A) enhances PM recruitment of PIP4K (Figs 4–6). Prior pulldown experiments of PIP5K and PIP4K from lysates required cross-linking the proteins, which might have occurred when the enzymes were simply colocalized on the PM rather than directly interacting (Wang et al., 2019). We therefore sought to distinguish between a direct PIP5K–PIP4K binding interaction versus PI(4,5)P_2_-induced co-enrichment on the PM. To this end, we devised an experiment whereby a bait protein (either PIP5K or control proteins) could be acutely localized to subdomains of the PM, with the same PI(4,5)P_2_ concentration. This was achieved using CID of baits with an endoplasmic reticulum (ER)-tethered protein, causing restricted localization of the bait protein to ER–PM contact sites – a subdomain of the PM (Fig. 7B). Enrichment of endogenous NG2–PIP4K2C at ER–PM contact sites was only observed when PIP5K1A was the bait; an unrelated peptide (myristoylated and palmitoylated peptide from Lyn kinase, Lyn_11_) or Mss4 did not enrich NG2–PIP4K2C (Fig. 7B). The use of Mss4 ruled out an effect of enhanced PI(4,5)P_2_ generation at contact sites, given that this enzyme increases PI(4,5)P_2_ as potently as PIP5K1A (Fig. 1A), yet does not cause recruitment of PIP4K2C.

Finally, we also demonstrate that PIP4K2A binding to PI(4,5)P_2_-containing supported lipid bilayers was greatly enhanced by addition of PIP5K to the membranes (Fig. 7C), but not by Mss4 (Fig. 7D). Clearly, PIP4K enzymes directly interact with PIP5Ks on PI(4,5)P_2_-containing lipid bilayers. The ability of PIP4K to bind to PIP5K on a PI(4,5)P_2_-containing bilayer also potentially explains the slightly accelerated initial rate of PI(4,5)P_2_ synthesis exhibited by PIP5K1A that we reported in Fig. 2C, given that PIP4K might initially introduce some avidity to the membrane interaction by PIP5K, before PI(4,5)P_2_ reaches a sufficient concentration that PIP4K-mediated inhibition is effective.

### Disruption of PI(4,5)P_2_ has differential effects on signaling

**Fig. 8.**
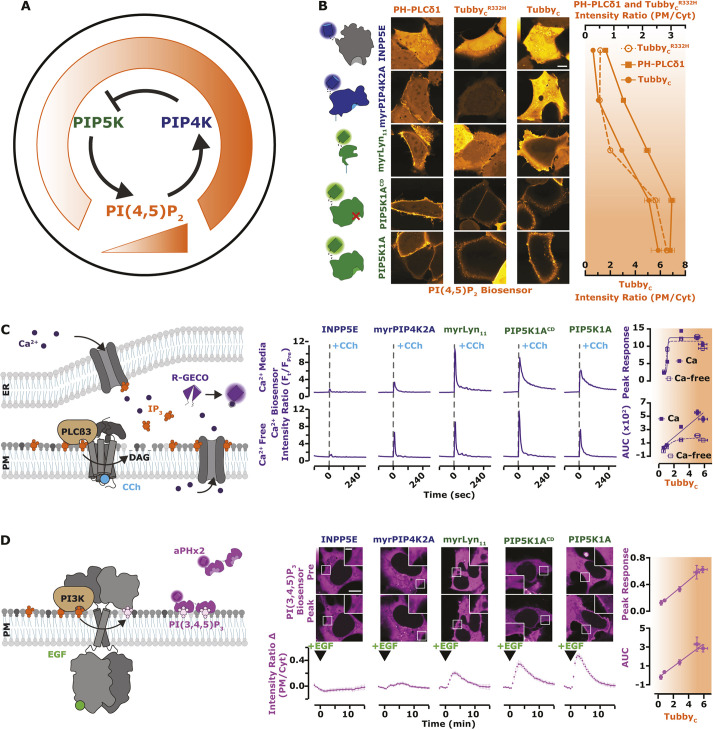
**PI3K, but not Ca^2+^**, **signaling is modulated across all concentration ranges of PI(4,5)P_2_.** (A) Proposed regulation of PIP5K by the low-affinity PI(4,5)P_2_ interaction with PIP4K. The working model for negative feedback of PIP5K via PIP4K resembles the thermostat regulation of temperature. When PI(4,5)P_2_ levels are high, PIP4K is recruited and held at the PM, via a direct low affinity interaction with PI(4,5)P_2_. At the PM, PIP4K interacts with and inhibits the catalytic activity of PIP5K, causing reduced PI(4,5)P_2_ synthesis. (B) PI(4,5)P_2_ biosensors detect a gradient of lipid levels. HEK293A cells were transfected with the indicated fluorescently tagged PI(4,5)P_2_ modulating proteins (INPP5E, myrPIP4K2A, myrLyn_11_, PIP5K1A catalytic dead or active) and the indicated PI(4,5)P_2_ biosensor (PH-PLCδ1, Tubby or Tubby^R332H^ displayed in orange) for 16–24 h. Scale bar: 10 µm. Mean±s.e.m. fluorescence intensity (PM/Cyt) from >120 cells imaged across three independent experiments is shown as points. (C) PLC-mediated Ca^2+^ signals saturate at tonic PI(4,5)P_2_ levels. Cartoon schematics of PLC mediated Ca^2+^ signaling and detection. HEK293A cells were transfected with the indicated fluorescently tagged construct and the Ca^2+^ sensor R-GECO (purple). During time-lapse confocal microscopy [performed with either compete imaging medium containing 1.8 mM Ca^2+^ (Ca^2+^ Media) or Ca^2+^-free Ringer's medium (Ca^2+^ Free)], cells were stimulated with 100 µM CCh as indicated. Traces represent the peak response of mean±s.e.m. change in fluorescence intensity (*F*_t_/*F*_Pre_ normalized to pre-stimulation levels) for >100 cells imaged across a minimum of three independent experiments. The peak response and total area under the curve (AUC) were plotted against the normalized ratio of Tubby_C_. (D) PI3K-mediated PI(3,4,5)P_3_ synthesis is linearly dependent on PI(4,5)P_2_ levels. Cartoon schematics show PI3K-mediated signaling and detection of PI(3,4,5)P_3_ upon the addition of EGF. HEK293A cells were transfected with the indicated fluorescently tagged construct and the PI(3,4,5)P_3_ biosensor, PH-ARNO^2G-I303Ex2^ (aPHx2) (magenta). Scale bars: 10 µm (main image), inset (2.5 µm). During time-lapse confocal microscopy, cells were stimulated with 10 ng/ml EGF, as indicated. Traces represent the peak response of mean±s.e.m. change in fluorescence intensity (change in PM/Cyt from pre-stimulation levels) of 35 cells imaged across a minimum of three independent experiments. The peak response and AUC were plotted against the normalized ratio of Tubby_C_.

Synthesizing all of these observations, we propose a simple homeostatic feedback loop that maintains PI(4,5)P_2_ levels in the PM (Fig. 8A) – when PI(4,5)P_2_ levels increase, PIP4K is recruited to the PM in sufficient quantities to inhibit PIP5K, halting further PI(4,5)P_2_ synthesis. If PI(4,5)P_2_ levels fall, PIP4K is one of the first PI(4,5)P_2_-binding proteins to be released (due to its low affinity), causing disinhibition of PIP5K and recovery of PI(4,5)P_2_. We next sought to test how perturbations of this homeostat would affect physiological function. We could produce graded changes in resting PI(4,5)P_2_ levels by overexpression of various components of the homeostat: enhanced PIP5K1A expression, either catalytically active or inactive, increases PI(4,5)P_2_; a myristoylated PIP4K2A retains PM localization even at low PI(4,5)P_2_, causing sustained reductions in PI(4,5)P_2_; and a PM-localized PI(4,5)P_2_ 5-OH phosphatase causes near complete ablation of the lipid. These constructs all show the expected changes in PM PI(4,5)P_2_ compared to a control, reported by three different PI(4,5)P_2_ biosensors. Of these, Tubby_c_ showed the largest degree of change in PM localization across all changes in PI(4,5)P_2_ levels (Fig. 8B). We then used these graded changes in steady-state PM PI(4,5)P_2_ to investigate the concentration requirements for the lipid in signaling.

PI(4,5)P_2_ is the substrate for PLC, the enzyme that cleaves it into second messengers diacylglycerol and inositol (1,4,5)-trisphosphate (IP_3_), triggering Ca^2+^ release from ER stores (Fig. 8C). Ca^2+^ release was indeed reduced by lower PI(4,5)P_2_ levels, but appeared to be maximal at tonic PI(4,5)P_2_ levels; it was unaffected by increased PM PI(4,5)P_2_. This was true for both peak Ca^2+^ release and total release from stores (assessed by measuring activity in calcium-free medium, Fig. 8C). Influx of extracellular Ca^2+^ was increased by elevated PI(4,5)P_2_ levels (Fig. 8C), consistent with a prior report that store-operated Ca^2+^ entry is enhanced by increased PIP5K activity (Chen et al., 2017). However, IP_3_-triggered Ca^2+^ release appears saturated at resting PI(4,5)P_2_. This strongly contrasts with the effects on another PI(4,5)P_2_ signaling pathway, class I phosphoinositide 3-OH kinase (PI3K). Epidermal growth factor (EGF) receptor stimulation activates PI3K, which converts a small fraction of PI(4,5)P_2_ into PIP_3_ (Fig. 8D). Using a sensitive PIP_3_ biosensor, we observed PIP_3_ production changing proportionately with PI(4,5)P_2_, never reaching a saturated level (Fig. 8D). PI3K activation therefore, unlike PLC, is sensitive to upregulation by alterations in PI(4,5)P_2_ homeostasis that enhance steady-state levels of the lipid, e.g. by enhanced PIP5K1A expression.

## DISCUSSION

The work presented herein reveals a remarkably simple homeostatic mechanism for PM PI(4,5)P_2_ levels (Fig. 8A). Here, the PIP4K family of enzymes serve as both receptor and control center, detecting PI(4,5)P_2_ and controlling the activity of the effector, PIP5K. This mechanism is also complementary to a previously identified homeostatic feedback, whereby PI4P catabolism is inactivated in cells until sufficient PI(4,5)P_2_ has been generated (Sohn et al., 2018). By these mechanisms, cells can ensure adequate PI(4,5)P_2_ is generated to support the cytoskeletal assembly, small solute transport, ion flux, membrane traffic and cell signaling processes controlled by PI(4,5)P_2_. The low affinity of PIP4K for PI(4,5)P_2_, and its and highly co-operative binding, makes PIP4Ks an excellent sensor for tonic PI(4,5)P_2_ levels. PIP4Ks are poised to sense PI(4,5)P_2_ generated in excess of the needs of the legion effector proteins for the lipids, ensuring these needs are met but not exceeded. Nevertheless, the relatively low PIP4K copy number of ∼2.5×10^5^ molecules per cell (Cho et al., 2022) is a small fraction of the total PI(4,5)P_2_ pool, estimated to be ∼10^7^ molecules (Wills and Hammond, 2022), ensuring little impact on the capacity of the lipid to interact with its effectors.

Since this paper was initially submitted for publication, another study has reported a similar homeostatic feedback loop in *Drosophila* photoreceptors, utilizing the fly homologue of septin 7 as the receptor and control center (Kumari et al., 2022). This conclusion is based on the observation that cells with reduced septin 7 levels have enhanced PIP5K activity in lysates, and exhibit more rapid PI(4,5)P_2_ resynthesis after PLC activation. However, changes in septin 7 membrane localization in response to acute alterations in PI(4,5)P_2_ levels, as well as direct interactions between PIP5K and septin 7, have yet to be demonstrated. Nevertheless, septin 7 has distinct properties as a potential homeostatic mediator; as a foundational member of the septin family, it is essential for generating all major types of septin filament (Spiliotis and Nakos, 2021). Therefore, a null allele for this subunit is expected to reduce the prevalence of the septin cytoskeleton by half. Given that septin subunits are found in mammalian cells at high copy number, around ∼10^6^ each (Cho et al., 2022), and the fact that septins bind PI4P and PI(4,5)P_2_ (Tanaka-Takiguchi et al., 2009; Zhang et al., 1999), it is likely that septin filaments sequester a significant fraction of the PM PI4P and PI(4,5)P_2_ through high-avidity interactions. In addition, membrane-bound septins appear to be effective diffusion barriers to PI(4,5)P_2_ and other lipids (Pacheco et al., 2022). We therefore speculate that septins might play a unique role in systems such as the fly photoreceptor with extremely high levels of PLC-mediated PI(4,5)P_2_ turnover. In such systems, the septin cytoskeleton can act as a significant buffer for PI4P and PI(4,5)P_2_, as well as corralling pools of the lipids for use at the rhabdomeres where the high rate of turnover occurs. This is in contrast to the role played by the PIP4Ks, where PI(4,5)P_2_ levels are held in a narrow range under conditions of more limited turnover, as found in most cells.

That PIP4K has such a crucial function for which catalytic activity is entirely dispensable is surprising. PIP4K catalytic activity varies among paralogs by almost four orders of magnitude (Clarke and Irvine, 2013); nevertheless, the ability of the enzymes to phosphorylate PI5P is known to be crucial for many of its other physiological functions (Poli et al., 2021; Ravi et al., 2021). However, the low-affinity PM PI(4,5)P_2_ binding that we describe, and its inhibition of PIP5K described previously (Wang et al., 2019), explain why PIP4Ks are expressed in cells in excess of PIP5K by as much as 10:1 (Cho et al., 2022; Geiger et al., 2012). This fact does not make sense relative to the catalytic activity of the enzymes, given that substrate of PIP4Ks, PI5P, is outnumbered by PI4P by ∼100-fold (Sarkes and Rameh, 2010).

Curiously, although phosphatidylinositol phosphate kinases are found throughout eukaryotes, PIP4Ks are limited to holozoa (animals and closely related unicellular organisms) (Khadka and Gupta, 2019). Indeed, we found the PIP5K from the fission yeast, *Saccharomyces cerevisiae*, does not interact with human PIP4Ks (Fig. 7) and cannot modulate PI(4,5)P_2_ levels in human cells without its catalytic activity (Fig. 1). This begs the question: how do *S. cerevisiae* regulate their own PI(4,5)P_2_ levels? Intriguingly, they seem to have a paralogous homeostatic mechanism: the dual PH domain containing protein Opy1 serves as receptor and control center (Ling et al., 2011), in an analogous role to PIP4K. Given that there is no mammalian homolog of Opy1, this homeostatic mechanism appears to have appeared at least twice through convergent evolution. Combined with hints of a role for septins in maintaining PI(4,5)P_2_ levels (Kumari et al., 2022), the possibility arises that there might yet be more feedback controls of PI(4,5)P_2_ levels to be discovered.

Despite minor differences in the ability of overexpressed PIP5K paralogs to recruit overexpressed PIP4K enzymes (Fig. 7A), we observed major differences in the ability of PIP4K paralogs to inhibit PI(4,5)P_2_ synthesis when over-expressed alone (Fig. 1C) or in combination with PIP5K (Fig. 2B). It is unclear what drives the partially overlapping inhibitory activity, where each PIP5K paralog can be attenuated by two or three PIP4Ks. This is however reminiscent of the biology of the PIPKs, where there is a high degree of redundancy among them, with few unique physiological functions assigned to specific paralogs (Burke et al., 2022). There might be hints of paralog-specific functions in our data; for example, enhanced PI(4,5)P_2_ induced by overexpressed PIP5K1C is only really attenuated by PIP4K2C (Fig. 2B). This could imply a requirement for PIP4K2C in regulating PI(4,5)P_2_ levels during PLC-mediated signaling, given the unique requirements for PIP5K1C in this process (Legate et al., 2012; Wang et al., 2004). Regardless, a full understanding of paralog selectivity will need to be driven by a detailed structural analysis of the interaction between PIP4Ks and PIP5Ks – which is not immediately apparent from their known crystal structures, especially given that PIP4Ks and PIP5Ks employ separate and distinct dimerization interfaces (Burke et al., 2022).

The apparently linear dependence of PI3K on available PI(4,5)P_2_ that we revealed after modulating PI(4,5)P_2_ homeostasis (Fig. 8) explains the enhanced PI3K signaling reported in PIP4K-null cells (Sharma et al., 2019; Wang et al., 2019). Intriguingly, PIP4Ks were reported to inhibit PI3K/Akt signaling two decades ago, but the mechanism was proposed to be through removal of its PI5P substrate, which was thought to somehow enhance accumulation of the PI3K lipid products, PIP_3_ and PI(3,4)P_2_ (Carricaburu et al., 2003). The key evidence that it was PI5P that caused the PI3K lipid accumulation came from the observation that it could be recapitulated by the *Shigella flexneri* effector protein IpgD, which generates some PI5P from PI(4,5)P_2_; this and the analogous *Salmonella* effector SopB both activate the PI3K/Akt pathway (Carricaburu et al., 2003; Marcus et al., 2001; Pendaries et al., 2006). However, it was recently shown that both SopB and IpgD are in fact novel phosphotransferases that directly convert PI(4,5)P_2_ into the PI3K signaling lipid PI(3,4)P_2_, explaining how these enzymes activate Akt (Walpole et al., 2022). It therefore seems more likely that PI(4,5)P_2_ downregulation is the most likely explanation for PI3K/Akt pathway inhibition by PIP4Ks.

In conclusion, our results reveal a simple yet elegant homeostatic mechanism that controls PM PI(4,5)P_2_ levels (Fig. 8A). Perturbation of this homeostasis reveals different sensitivities of PLC and PI3K signaling, with the latter showing enhanced activation with elevated PI(4,5)P_2_. This likely explains why the PI3K, and not the PLC pathway, drives the phenotype of PIP4K-null fruit flies (Sharma et al., 2019). More broadly, such differences in the sensitivity of PI(4,5)P_2_-dependent PM functions to lipid concentration might go a long way in explaining the phenotypic diversity of diseases associated with dysregulated PI(4,5)P_2_ metabolism. For example, they might explain why a selective inhibitor of PI3Kα can correct aberrant kidney function associated with Lowe syndrome models (Berquez et al., 2020). Indeed, experimental manipulation of PI(4,5)P_2_ homeostasis will now afford the ability to determine which of the panoply of PI(4,5)P_2_-dependent PM functions are dysregulated by pathological alterations – perhaps bringing novel therapeutic targets into view.

## MATERIALS AND METHODS

### Cell culture and lipofection

HeLa (ATCC CCL-2) and HEK293A (Thermo Fisher Scientific R705-07) cells were cultured in DMEM (low glucose; Life Technologies 10567022) supplemented with 10% heat-inactivated fetal bovine serum (Life Technologies 10438-034), 100 units/ml penicillin, 100 µg/ml streptomycin (Life Technologies 15140122) and 1:1000 chemically defined lipid supplement (Life Technologies 11905031) at 37°C with a humidified atmosphere with 5% CO_2_. Cells were passaged twice per week diluting 1 in 5 after dissociation in TrpLE (Life Technologies 12604039). HEK293A cells with endogenous PIP4K2 paralog alleles tagged with split NeonGreen2 (NG2) were generated similarly to described previously (Leonetti et al., 2016) using a protocol from Zewe et al. (2018). In brief, Platinum Cas9 (Thermo Fisher Scientific B25640) was precomplexed with gRNA and electroporated into HEK293^NG2-1-10^ cells in combination with a single-stranded HDR Template (IDT). Sequences are provided in Table S9. The HDR template contains 70 bp homology arms, the NG2-11 sequence, and a flexible linker in frame with the appropriate PIP4K paralog: PIP4K2A and PIP4K2B (5′-CATCATATCGGTAAAGGCCTTTTGCCACTCCTTGAAGTTGAGCTCGGTACCACT TCCTGGACCTTGAAACAAAACTTCCAATCCGCCACC-3′) and PIP4K2C (5′-ATGACCGAGCTCAACTTCAAGGAGTGGCAAAAGGCCTTTACCGATATGATGGGTGGCGGC-3′). After recovery, FACS (University of Pittsburgh Flow Cytometry Core) was used to sort NG2-positive cells. These NG2-PIP4K2A, PIP4K2B and PIP4K2C cells were cultured under identical conditions to the HeLa and HEK293A cells.

### Chemicals and reagents

Rapamycin (Thermo Fisher Scientific BP2963-1) was dissolved in DMSO at 1 mM and stored as a stock at −20°C, it was used in cells at 1 µM. EGTA (VWR EM-4100) was dissolved in water at 0.5 M and stored at room temperature, it was used in cells at 5 mM. EGF (Corning CB-40052) was dissolved in water at 100 µg/ml and stored as a stock at −20°C, it was used in cells at 10 ng/ml. Carbachol (Thermo Fisher Scientific AC10824-0050) was dissolved in water at 50 mM and stored as a stock at −20°C, it was used in cells at 100 µM. Atropine (Thermo Fisher Scientific AC226680100) was dissolved in 100% ethanol at 25 mM and stored as a stock at −20°C, it was used in cells at 5 µM.

### Plasmids and cloning

The EGFP (*Aequorea victoria* GFP containing F64L and S65T mutations; Cormack et al., 1996), mCherry (*Discoma* DsRed monomeric variant; Shaner et al., 2004), mTagBFP2 (*Entacmaea quadricolor* protein eqFP578; Subach et al., 2011), iRFP713 [*Rhodopseudomonas palustris* (Rp) bacteriophytochrome BphP2; Filonov et al., 2011] and iRFP670 (RpBphP6 iRFP702 containing V112I, K174M and I247C mutations; Shcherbakova and Verkhusha, 2013) fluorophores were used in the Clontech pEGFP-C1, -C2, and -N1 backbones as described previously (Zewe et al., 2018). Mutated constructs were generated using site-directed mutagenesis using targeted pairs of DNA oligonucleotides, which were custom made and supplied by Thermo Fisher Scientific. New plasmids used in this study were generated using standard restriction-ligation or by using NEBuilder HiFi DNA Assembly (New England Biolabs E552OS). *Homo sapiens* (Hs)PIP5K1A, HsPIP5K1B, yeast Mss4 and HsPIP4K2C were obtained as human codon optimized synthetic gBlocks (IDT). Otherwise, plasmids were obtained from the sources listed in Table S8. All constructs were sequence verified using Sanger DNA sequencing. Plasmids constructed for this study are available through Addgene (see Table S8).

### Purification of PIP5K1A and Mss4

Gene sequences encoding human PIP5K1A and yeast Mss4 kinase domain were cloned into a FastBac1 vector to create the following vectors: His6-MBP-TEV-(Gly)5-PIP5K1A [amino acids (aa) 1–546] and His6-MBP-TEV-(Gly)5-Mss4 (aa 379–779). BACMIDs and baculovirus were generated as previously described (Hansen et al., 2019). ES-Sf9 cells were infected with baculovirus using an optimized multiplicity of infection (MOI), typically 2% v/v, which was empirically determined from small-scale test expression. Infected cells were typically grown for 48 h at 27°C in ESF 921 Serum-Free Insect Cell Culture medium (Expression Systems, Cat# 96-001-01) and then harvested by centrifugation. Insect cell pellets were then washed with 1× PBS (pH 7.2) and centrifuged (2200 ***g*** for 10 min). The final cell pellet was combined with an equal volume of buffer containing 1× PBS pH 7.2, 10% glycerol and 2× Sigma protease inhibitor cocktail tablet solution before transferring to the −80°C freezer for storage. For purification, frozen cells were thawed in an ambient water bath and then resuspended in buffer containing 50 mM Na_2_HPO_4_ pH 8.0, 10 mM imidazole, 400 mM NaCl, 5% glycerol, 1 mM PMSF, 5 mM 2-mercaptoethanol (BME), 100 µg/ml DNase and 1× Sigma protease inhibitor cocktail tablet. Cells were lysed using a glass dounce homogenizer. Lysate was then centrifuged at 35,000 rpm (140,000 ***g***) for 60 min in a Beckman Ti-45 rotor at 4°C. High speed supernatant was combined with 6 ml of Ni-NTA Agarose (Qiagen, Cat# 30230) and stirred in a beaker for 1–2 h at 4°C. Following batch binding, resin was collected in 50 ml tubes, centrifuged (2200 ***g*** for 10 min), and washed with buffer containing 50 mM Na_2_HPO_4_ pH 8.0, 10 mM imidazole, 400 mM NaCl and 5 mM BME. Ni-NTA resin with His6-MBP-(Asn)10-TEV-(Gly)5-PIP5K1A bound was washed in a gravity flow column with 100 ml of 50 mM Na_2_HPO_4_ pH 8.0, 30 mM imidazole, 400 mM NaCl, 5% glycerol and 5 mM BME buffer. Protein elution was achieved by washing the resin with buffer containing 50 mM Na_2_HPO_4_ pH 8.0, 500 mM imidazole, 400 mM NaCl, 5% glycerol and 5 mM BME. Peak fractions were pooled, combined with 200 µg/ml His6-TEV(S291V) protease, and dialyzed against 4 l of buffer containing 20 mM Tris-HCl pH 8.0, 200 mM NaCl and 2.5 mM BME for 16-18 h at 4°C. The next day, dialysate was combined 1:1 by volume with 20 mM Tris-HCl pH 8.0, 1 mM TCEP to reduce the NaCl to a final concentration of 100 mM. Precipitate was removed by centrifugation (2200 ***g*** for 10 min) and a 0.22 µm syringe filtration. Clarified dialysate was bound to a MonoS cation exchange column (GE Healthcare, Cat# 17-5168-01) equilibrated with buffer containing 20 mM Tris-HCl pH 8.0, 100 mM NaCl and 1 mM TCEP. Proteins were resolved over a 10–100% linear gradient (0.1–1M NaCl, 45 CV, 45 ml total, 1 ml/min flow rate). (Gly)_5_–PIP5K1A and (Gly)_5_–Mss4 eluted from the MonoS in the presence of 375–450 mM NaCl. Peak fractions containing PIP5K1A were pooled, concentrated in a 30 kDa MWCO Vivaspin 6 centrifuge tube (GE Healthcare, Cat# 28-9323-17), and loaded onto a 24 ml Superdex 200 10/300 GL (GE Healthcare, Cat# 17-5174-01) size exclusion column equilibrated in 20 mM Tris-HCl pH 8.0, 200 mM NaCl, 10% glycerol and 1 mM TCEP. Peak fractions were concentrated to 10–50 µM using a 30 kDa MWCO Amicon centrifuge tube (Millipore Sigma) before snap freezing with liquid nitrogen. PIP5K1A and Mss4 were stored in −80°C as single-use aliquots.

### Purification of PIP4K2A

The gene encoding human PIP4K2A was cloned into a pETM-derived bacterial expression vector (EMBL Protein Expression and Purification Core Facility) to create the following fusion protein: His6-SUMO3-(Gly)5-PIP4K2A (aa 1–406). Recombinant PIP4KA was expressed in BL21 (DE3) Star *Escherichia coli* (MacroLab protein expression facility at UC Berkeley; which lack endonuclease for increased mRNA stability). Using 4 l of Terrific Broth, bacterial cultures were grown at 37°C until the optical density at 600 nm (OD_600_)=0.6. Cultures were then shifted to 18°C for 1 h to cool down. Protein expression was induced with 50 µM IPTG and bacteria were allowed to express protein for 20 h at 18°C before being harvested by centrifugation (2200 ***g*** for 10 min). For purification, cells were lysed into buffer containing 50 mM Na_2_HPO_4_ pH 8.0, 400 mM NaCl, 0.4 mM BME, 1 mM PMSF (added twice, 15 min intervals), 100 µg/ml DNase and 1 mg/ml lysozyme using a microtip sonicator. Lysate was centrifuged at 16,000 rpm (35,172 ***g***) for 60 min in a Beckman JA-17 rotor chilled to 4°C. Lysate was circulated over a 5 ml HiTrap Chelating column (GE Healthcare, Cat# 17-0409-01) that had been equilibrated with 100 mM CoCl_2_ for 1 h, washed with MilliQ water, and followed by buffer containing 50 mM Na_2_HPO_4_ pH 8.0, 400 mM NaCl and 0.4 mM BME. Recombinant PIP4K2A was eluted with a linear gradient of imidazole (0­–500 mM, 8 CV, 40 ml total, 2 ml/min flow rate). Peak fractions were pooled, combined with 50 µg/ml of His6–SenP2 (SUMO protease), and dialyzed against 4 l of buffer containing 25 mM Na_2_HPO_4_ pH 8.0, 400 mM NaCl, and 0.4 mM BME for 16–18 h at 4°C. Following overnight cleavage of the SUMO3 tag, dialysate containing His6–SUMO3, His6–SenP2 and (Gly)_5_–PIP4K2A was recirculated for at least 1 h over a 5 ml HiTrap(Co^2+^) chelating column. Flow-through containing (Gly)_5_–PIP4K2A was then concentrated in a 30 kDa MWCO Vivaspin 6 before loading onto a Superdex 200 size exclusion column equilibrated in 20 mM HEPES pH 7, 200 mM NaCl, 10% glycerol, 1 mM TCEP. In some cases, cation exchange chromatography was used to increase the purity of (Gly)_5_–PIP4K2A before loading on the Superdex 200. In those cases, we equilibrated a MonoS column with 20 mM HEPES [pH 7], 100 mM NaCl, 1 mM TCEP buffer. PIP4K2A (pI=6.9) bound to the MonoS was resolved over a 10–100% linear gradient (0.1–1 M NaCl, 30 CV, 30 ml total and 1.5 ml/min flow rate). Peak fractions collected from the Superdex 200 were concentrated in a 30 kDa MWCO Amicon centrifuge tube and snap frozen at a final concentration of 20-80 µM using liquid nitrogen.

### Purification of PH-PLCδ1 domain

The coding sequence of human PH-PLCδ1 (aa 11–140) was expressed in BL21 (DE3) *E. coli* as a His6–SUMO3–(Gly)_5_–PLCδ1 (aa 11–140) fusion protein. Bacteria were grown at 37°C in Terrific Broth to an OD_600_ of 0.8. Cultures were shifted to 18°C for 1 h, induced with 0.1 mM IPTG, and allowed to express protein for 20 h at 18°C before being harvested. Cells were lysed into 50 mM Na_2_HPO_4_ pH 8.0, 300 mM NaCl, 0.4 mM BME, 1 mM PMSF and 100 µg/ml DNase using a microfluidizer. Lysate was then centrifuged at 16,000 rpm (35,172 ***g***) for 60 min in a Beckman JA-17 rotor chilled to 4°C. Lysate was circulated over 5 ml HiTrap Chelating column (GE Healthcare, Cat# 17-0409-01) charged with 100 mM CoCl_2_ for 1 h. Bound protein was then eluted with a linear gradient of imidazole (0–500 mM, 8 CV, 40 ml total, 2 ml/min flow rate). Peak fractions were pooled, combined with SUMO protease (50 µg/ml final concentration), and dialyzed against 4 l of buffer containing 50 mM Na_2_HPO_4_ pH 8.0, 300 mM NaCl, and 0.4 mM BME for 16–18 h at 4°C. Dialysate containing SUMO cleaved protein was recirculated for 1 h over a 5 ml HiTrap Chelating column. Flow-through containing (Gly)_5_–PLCδ1 (aa 11–140) was then concentrated in a 5 kDa MWCO Vivaspin 20 before being loaded on a Superdex 75 size exclusion column equilibrated in 20 mM Tris-HCl pH 8.0, 200 mM NaCl, 10% glycerol and 1 mM TCEP. Peak fractions containing (Gly)_5_–PLCδ1 (aa 11–140) were pooled and concentrated to a maximum of 75 µM (1.2 mg/ml) before freezing in liquid nitrogen.

### Purification of OCRL

The coding sequence of human 5-phosphatase OCRL (aa 234–539 of a 901 aa isoform) was expressed in BL21 (DE3) *E. coli* as a His6-MBP-(Asn)10-TEV-(Gly)5-OCRL fusion protein. Bacteria were grown at 37°C in Terrific Broth to an OD600 of 0.8. Cultures were shifted to 18°C for 1 h, induced with 0.1 mM IPTG, and allowed to express protein for 20 h at 18°C before being harvested. Cells were lysed into 50 mM Na H_2_PO_4_ pH 8.0, 300 mM NaCl, 0.4 mM BME, 1 mM PMSF and 100 µg/ml DNase using a microfluidizer. Lysate was then centrifuged at 16,000 rpm (35,172 ***g***) for 60 min in a Beckman JA-17 rotor chilled to 4°C. Lysate was circulated over 5 ml HiTrap Chelating column (GE Healthcare, Cat# 17-040901) charged with 100 mM CoCl_2_ for 1 h. Bound protein was eluted with a linear gradient of imidazole (0–500 mM, 8 CV, 40 ml total, 2 ml/min flow rate). Peak fractions were pooled, combined with TEV protease (75 µg/ml final concentration), and dialyzed against 4 l of buffer containing 50 mM NaH_2_PO_4_ pH 8.0, 300 mM NaCl and 0.4 mM BME for 16–18 h at 4°C. Dialysate containing TEV protease cleaved protein was recirculated for 1 h over a 5 ml HiTrap Chelating column. Flow-through containing (Gly)_5_ protein was then concentrated in a 5 kDa MWCO Vivaspin 20 before being loaded on a Superdex 75 (10/300 GL) size exclusion column equilibrated in 20 mM Tris-HCl pH 8.0, 200 mM NaCl, 10% glycerol and 1 mM TCEP. Peak fractions were pooled and concentrated before snap freezing in liquid nitrogen.

### Sortase-mediated peptide ligation

PIP4K2A, PIP5K1A and PH-PLCδ1 were labeled on a N-terminal (Gly)5 motif using sortase-mediated peptide ligation (Guimaraes et al., 2013; Hansen et al., 2019). Initially, a LPETGG peptide was labeled with either Alexa Fluor 488, Alexa Fluor 647 or Cy5 conjugated to an amine reactive N-hydroxysuccinimide (NHS) (e.g. NHS–Alexa488). Protein labeling was achieved by combining the fluorescently labeled LPETGG peptide with the following reagents: 50 mM Tris-HCl pH 8.0, 150 mM NaCl, 50 µM (Gly)_5_-protein, 500 µM Alexa488–LPETGG and 10–15 µM His6–sortase. This reaction mixture was incubated at 16–18°C for 16–20 h, before buffer exchange with a G25 Sephadex column (e.g. PD10) to remove the majority of dye and dye-peptide. The His6–sortase was then captured on Ni-NTA agarose resin (Qiagen) and unbound labeled protein was separated from remaining fluorescent dye and peptide using a Superdex 75 or Superdex 200 size exclusion column (24 ml bed volume).

### Preparation of small unilamellar vesicles

The following lipids were used to generate small unilamellar vesicles (SUVs): 1,2-dioleoyl-sn-glycero-3-phosphocholine (18:1 DOPC, Avanti #850375C), L-α-phosphatidylinositol-4-phosphate [Brain PI(4)P, Avanti #840045X], L-α-phosphatidylinositol-4,5-bisphosphate [Brain PI(4,5)P_2_, Avanti #840046X] and 1,2-dioleoyl-sn-glycero-3-phospho-L-serine (18:1 DOPS, Avanti #840035C). Lipids were purchased as single-use ampules containing between 0.1–5 mg of lipids dissolved in chloroform. Brain PI(4)P and PI(4,5)P_2_ were purchased as 0.25 mg/ml stocks dissolved in chloroform:methanol:water (20:9:1). To make liposomes, 2 µmoles total lipids were combined in a 35 ml glass round bottom flask containing 2 ml of chloroform. Lipids were dried to a thin film using rotary evaporation with the glass round-bottom flask submerged in a 42°C water bath. After evaporating all the chloroform, the round bottom flask was flushed with nitrogen gas for at least 30 min. We resuspended the lipid film in 2 ml of PBS pH 7.2, making a final concentration of 1 mM total lipids. All lipid mixtures expressed as percentages [e.g. 98% DOPC, 2% PI(4)P] are equivalent to molar fractions. For example, a 1 mM lipid mixture containing 98% DOPC and 2% PI(4)P is equivalent to 0.98 mM DOPC and 0.02 mM PI(4)P. To generate 30–50 nm SUVs, 1 mM total lipid mixtures were extruded through a 0.03 µm pore size 19 mm polycarbonate membrane (Avanti #610002) with filter supports (Avanti #610014) on both sides of the PC membrane. Hydrated lipids at a concentration of 1 mM were extruded through the PC membrane 11 times.

### Preparation of supported lipid bilayers

SLBs were formed on 25×75 mm coverglass (IBIDI, #10812). Coverglass was first cleaned with 2% Hellmanex III (Thermo Fisher Scientific, cat. #14-385-864) heated to 60–70°C in a glass coplin jar and incubated for at least 30 min. We washed the coverglass extensively with MilliQ water and then etched with Pirahna solution (1:3, hydrogen peroxide:sulfuric acid) for 10–15 min the same day SLBs were formed. Etched coverglass, in water, was rapidly dried with nitrogen gas before adhering to a six-well sticky-side chamber (ibidi, cat. #80608). SLBs were formed by flowing 30 nm SUVs diluted in PBS (pH 7.2) to a total lipid concentration of 0.25 mM. After 30 min, IBIDI chambers were washed with 5 ml of PBS (pH 7.2) to remove non-absorbed SUVs. Membrane defects were blocked for 15 min with a 1 mg/ml β-casein (Thermo Fisher Scientific, #37528) diluted in 1× PBS (pH 7.4). Before use as a blocking protein, frozen 10 mg/ml β-casein stocks were thawed, centrifuged for 30 min at 21,370 ***g*** and 0.22 µm syringe filtered. After blocking SLBs with β-casein, membranes were washed again with 1 ml of PBS, followed by 1 ml of kinase buffer before TIRFM.

### Microscopy

For all live-cell imaging experiments, cells were imaged in 1.6 ml of experiment specific imaging medium. Base imaging medium contained FluoroBrite DMEM (Life Technologies A1896702) supplemented with 25 mM HEPES (pH 7.4) and 1:1000 chemically defined lipid supplement (SF CHIM). The medium was then further supplemented with either 10% fetal bovine serum (CHIM) or 0.1% BSA (0.1% BSA CHIM). Alternatively, Ca^2+^-free Ringer's solution (Ca^2+^ Free) was used, containing 160 mM NaCl, 2.5 mM KCl, 1 mM MgCl_2_, 8 mM glucose and 10 mM NaHEPES, pH 7.5. For treatments, 0.4 ml of experiment specific imaging medium containing fivefold final concentration of compound was applied to the dish (or 0.5 ml for a second addition).

Confocal imaging was performed on a Nikon TiE A1R platform with acquisition in resonant mode with a 100×1.45 NA plan-apochromatic objective. The signal-to-noise ratio was improved by taking 8 or 16 frame averages. Excitation of fluorophores was accomplished using a dual fiber-coupled LUN-V laser launch with 405-nm (BFP), 488-nm (EGFP and NG2), 561-nm (mCherry) and 640-nm (iRFP) lines. Emission was collected on four separate photomultiplier tubes with blue (425-475 nm), green (500-550 nm), yellow/orange (570-620 nm), and far-red (663-737 nm) filters. Blue and yellow/orange channels were recorded concurrently, as were green and far-red. The confocal pinhole was defined as 1.2× the Airy disc size of the longest wave-length channel used in the experiment. In some instances, Nikon Elements denoising software was used to further enhance the signal-to-noise ratio.

For TIRFM and single-molecule imaging (SMol), a separate Nikon TiE platform coupled with a Nikon TIRF illuminator arm and 100×1.45 NA plan-apochromatic objective was used. Excitation of fluorophores was accomplished using an Oxxius L4C laser launch with 405-nm (BFP), 488-nm (EGFP and NG2), 561-nm (mCherry), and 638-nm (iRFP) lines. Emission was collected through dual-pass filters (Chroma) with blue (420–480 nm) and yellow/orange (570–620 nm) together, and green (505–550 nm) and far-red (650–850 nm) together. An ORCA-Fusion BT sCMOS camera (Hamamatsu) was used to capture images. For TIRFM, images were captured with 2×2 pixel binning. For SMol, the NG2 channel was excited with 100% power for 50 ms from the 488-nm laser in a 16×16 µm region of the PM. Images were registered in rolling shutter mode with 2×2 pixel binning with a 1.5× magnifier lens.

For all types of imaging, Nikon Elements software was used to acquire all images for all experiments and all data was saved with the ND2 file extension.

Membrane binding and lipid phosphorylation reactions reconstituted on SLBs were visualized using an inverted Nikon Eclipse Ti2 microscope using a 100× Nikon (1.49 NA) oil immersion TIRF objective. TIRF microscopy images of SLBs were acquired using an iXion Life 897 EMCCD camera (Andor Technology Ltd., UK). Fluorescently labeled proteins were excited with either a 488 nm, 561 nm or 637 nm diode laser (OBIS laser diode, Coherent Inc. Santa Clara, CA, USA) controlled with a Vortran laser drive with acousto-optic tunable filters (AOTF) control. The power output measured through the objective for single particle imaging was 1–2 mW. Excitation light was passed through the following dichroic filter cubes before illuminating the sample: (1) ZT488/647rpc and (2) ZT561rdc (ET575LP) (Semrock). Fluorescence emission was detected on the iXion Life 897 EMCCD camera position after a Nikon emission filter wheel housing the following emission filters: ET525/50M, ET600/50M, ET700/75M (Semrock). All experiments were performed at room temperature (23°C). Microscope hardware was controlled by Nikon NIS elements.

### Image analysis

Analysis of all images was accomplished using Fiji software (Schindelin et al., 2012) using the LOCI BioFormats importer (Linkert et al., 2010). Custom macros were written to generate channel-specific montages displaying all *x,y* positions captured in an experiment in concatenated series (available upon request). In these montages, individual regions of interest (ROIs) were generated around displayed cells.

For confocal images, the ratio of fluorescence intensity between specific compartments was analyzed as described previously (Zewe et al., 2018). In brief, a custom macro was used to generate a compartment of interest specific binary mask through à trous wavelet decomposition (Olivo-Marin, 2002). This mask was applied to measure the fluorescence intensity within the given compartment while normalizing to the mean pixel intensity in the ROI. ROI corresponded to the whole cell (denoted as the PM/Cell ratio) or a region of cytosol (PM/Cyt), as indicated on the *y*-axis of individual figures.

For TIRFM images, a minimum intensity projection was used to generate ROIs within the smallest footprint of the cells. Background fluorescence was measured and subtracted from all images at all timepoints. The average pixel intensity in each frame (*F*_t_) was normalized to the mean pixel intensity in the ROI of the time points before treatment (*F*_pre_) to yield *F*_t_/*F*_pre_.

Quantitative data was imported into Prism 8 (GraphPad) for statistical analysis and the generation of graphs and plots. D'Agostino and Pearson normality tests showed data that significantly varied from normal distribution, data were then subjected to a nonparametric Kruskal–Wallis test. If significant difference was found between sample medians, a post hoc Dunn's multiple comparison test was run.

Representative images were selected based on fluorescence measurements near the median of the sampled population, displayed typical morphology, and robust signal-to-noise ratio. If adjusting brightness or contrast, any changes were made across the entire image.

### Single-molecule analysis using TrackMate

Mean photon count was estimated using Fiji (Schindelin et al., 2012). HEK293A cells expressing PH-PLCδ1–mNG2×1-3, NG2–PIP4K2A, NG2–PIP4K2B or NG2–PIP4K2C cells were imaged using SMol settings. Raw images were converted into 32-bit, background subtracted and gray levels converted into photon counts. These images were then run through Fiji using the TrackMate plugin. Settings for molecule localization were: LoG detector: estimated blob diameter 0.18 µm, threshold 40; initial thresholding by quality; filters on spots: total intensity to match surface localized particles, excluding puncta less than 3; simple LAP tracker: linking max distance 0.5 µm, gap-closing max distance 0.5 µm, gap-closing max frame gap 2. To determine fluorescence intensity per spot, histograms of mean intensity, in each condition, were generated using a 5-photon bin size.

### Kinetic measurements of PI(4,5)P_2_ production

The kinetics of PI(4)P phosphorylation was measured on SLBs formed in ibidi chambers and visualized using TIRFM as previously described (Hansen et al., 2019). Reaction buffer contained 20 mM HEPES pH 7.0, 150 mM NaCl, 1 mM ATP, 5 mM MgCl_2_, 0.5 mM EGTA, 20 mM glucose, 200 µg/ml β-casein (Thermo Fisher Scientific, #37528), 20 mM BME, 320 µg/ml glucose oxidase (Serva, #22780.01 *Aspergillus niger*), 50 µg/ml catalase (Sigma, #C40-100MG Bovine Liver) and 2 mM Trolox (UV treated; Hansen et al., 2019). Perishable reagents (i.e. glucose oxidase, catalase and Trolox) were added 5–10 min before image acquisition. For all experiments, we monitored the change in PI(4)P or PI(4,5)P_2_ membrane density using solution concentrations of 20 nM Alexa647–DrrA(544-647) or 20 nM Alexa488–PLCδ1, respectively.

## Supplementary Material

10.1242/joces.261494_sup1 Supplementary information
