# Supplementary material for: A novel homeostatic mechanism tunes PI(4,5)P2-dependent signaling at the plasma membrane
Source: J Cell Sci. 2023 Aug 29;136(16):jcs261494. doi: 10.1242/jcs.261494 (PMC10482388; doi:10.1242/jcs.261494)
Supplement: Supplementary information [file joces-136-261494-s1.pdf]

Table S1. P values from Kruskal-Wallis test with Dunn's correction for multiple comparisons for TubbycR332H ($PI(4,5)P_2$) biosensor data presented in Figure 1A. Significant variation was observed among groups by one-way ANOVA (KW statistic = 5089, P <0.0001). Significant results are highlighted in bold and n values are displayed.

Construct:	EGFP (n=2294)	Mss4	Mss4^{CD}	PIP5K1A	PIP5K1A^{CD}	PIP5K1B	PIP5K1B^{CD}	PIP5K1C
Mss4 (n=90)	**<0.0001**							
Mss4^{CD} (n=90)	**0.0324**	**<0.0001**						
PIP5K1A (n=299)	**<0.0001**	**<0.0001**	**<0.0001**					
PIP5K1A^{CD} (n=90)	**<0.0001**	**<0.0001**	**<0.0001**	>0.9999				
PIP5K1B (n=180)	**<0.0001**	**<0.0001**	**<0.0001**	>0.9999	>0.9999			
PIP5K1B^{CD} (n=90)	**<0.0001**	**<0.0001**	**0.0001**	>0.9999	>0.9999	>0.9999		
PIP5K1C (n=1729)	**<0.0001**	**0.0003**	**<0.0001**	>0.9999	0.4741	**0.0094**	0.2673	
PIP5K1C^{CD} (n=384)	**<0.0001**	**0.0347**	**<0.0001**	0.3338	0.0743	**0.001**	**0.0396**	>0.9999

Table S2. P values from Kruskal-Wallis test with Dunn's correction for multiple comparisons for P4Mx2 (PI4P) biosensor data presented in Figure 1B. Significant variation was observed among groups by one-way ANOVA (KW statistic = 231.2, P <0.0001). Significant results are highlighted in bold and n values are displayed.

Construct	EGFP (n=90)	Mss4	Mss4^{CD}	PIP5K1A	PIP5K1A^{CD}	PIP5K1B	PIP5K1B^{CD}	PIP5K1C
Mss4 (n=90)	**<0.0001**							
Mss4^{CD} (n=90)	>0.9999	**<0.0001**						
PIP5K1A (n=90)	**<0.0001**	**0.0325**	**<0.0001**					
PIP5K1A^{CD} (n=90)	**<0.0001**	>0.9999	**0.0004**	**<0.0001**				
PIP5K1B (n=90)	**<0.0001**	**0.0108**	**<0.0001**	>0.9999	**<0.0001**			
PIP5K1B^{CD} (n=90)	**<0.0001**	>0.9999	**<0.0001**	**0.0008**	>0.9999	**0.0002**		
PIP5K1C (n=90)	**<0.0001**	0.1166	**<0.0001**	>0.9999	**0.0001**	>0.9999	**0.004**	
PIP5K1C^{CD} (n=90)	**<0.0001**	>0.9999	**<0.0001**	0.6371	0.3208	0.2742	>0.9999	>0.9999

Journal of Cell Science • Supplementary information

Table S3. P values from Kruskal-Wallis test with Dunn's correction for multiple comparisons for Tubby$_c^{R332H}$ (PI(4,5)P_2) biosensor data presented in Figure 1C. Significant variation was observed among groups by one-way ANOVA (KW statistic = 231.1, P <0.0001). Significant results are highlighted in bold and n values are displayed above.

Construct	BFP (n=90)	INPP5E	PIP4K2A	PIP4K2A^{CD}	PIP4K2B	PIP4K2B^{CD}	PIP4K2C
INPP5E (n=90)	**<0.0001**						
PIP4K2A (n=90)	**<0.0001**	0.0032					
PIP4K2A^{CD} (n=90)	0.0658	**<0.0001**	**0.0159**				
PIP4K2B (n=90)	**<0.0001**	0.1877	>0.9999	**0.0001**			
PIP4K2B^{CD} (n=90)	>0.9999	**<0.0001**	**<0.0001**	0.1118	**<0.0001**		
PIP4K2C (n=90)	0.5904	**<0.0001**	**0.0008**	>0.9999	**<0.0001**	0.8988	
PIP4K2C^{CD} (n=90)	>0.9999	**<0.0001**	**<0.0001**	**0.0047**	**<0.0001**	>0.9999	0.0696

Table S4. P values from Kruskal-Wallis test with Dunn's correction for multiple comparisons for Tubby$_C^{R332H}$ (PI(4,5)P_2) biosensor data presented in Figure 2B. Significant variation was observed among groups by one-way ANOVA (FP KW statistic = 74.63, P <0.0001; PIP5K1A KW statistic = 86.53, P <0.0001; PIP5K1B KW statistic = 82.22, P <0.0001; PIP5K1C KW statistic = 23.94, P <0.0001). Significant results are highlighted in bold and n values are displayed.

EGFP	EGFP (n=90)	PIP4K2A	PIP4K2B
PIP4K2A (n=90)	**<0.0001**		
PIP4K2B (n=90)	**<0.0001**	0.8805	
PIP4K2C (n=90)	>0.9999	**<0.0001**	**<0.0001**
PIP5K1A	+BFP	+ PIP4K2A	+ PIP4K2B
+ PIP4K2A (n=90)	**<0.0001**		
+ PIP4K2B (n=90)	**<0.0001**	>0.9999	
+ PIP4K2C (n=90)	**<0.0001**	>0.9999	>0.9999
PIP5K1B	+BFP	+ PIP4K2A	+ PIP4K2B
+ PIP4K2A (n=90)	**<0.0001**		
+ PIP4K2B (n=90)	**<0.0001**	**0.0027**	
+ PIP4K2C (n=90)	0.7478	**0.0061**	**<0.0001**
PIP5K1C	+BFP	+ PIP4K2A	+ PIP4K2B
+ PIP4K2A (n=90)	>0.9999		
+ PIP4K2B (n=90)	0.0933	0.3609	
+ PIP4K2C (n=90)	**<0.0001**	**0.0007**	0.2853

Table S5. P values from Kruskal-Wallis test with Dunn's correction for multiple comparisons for indicated NG2-PIP4K paralog data presented in Figure 5B. Significant variation was observed among groups by one-way ANOVA (PIP4K2A KW statistic = 95.78, P <0.0001; PIP4K2B KW statistic = 25.09, P <0.0001; PIP4K2C KW statistic = 96.96, P <0.0001). Significant results are highlighted in bold and n values are displayed.

NG2-PIP4K2A		
	BFP (n=90)	Mss4
Mss4 (n=90)	**<0.0001**	
$Mss4^{CD}$ (n=90)	>0.9999	**<0.0001**

NG2-PIP4K2B		
	BFP (n=89)	Mss4
Mss4 (n=90)	**0.0313**	
$Mss4^{CD}$ (n=90)	**0.0448**	**<0.0001**

NG2-PIP4K2C		
	BFP (n=90)	Mss4
Mss4 (n=88)	**<0.0001**	
$Mss4^{CD}$ (n=90)	>0.9999	**<0.0001**

Table S6. P values from Kruskal-Wallis test with Dunn's correction for multiple comparisons for PIP5K paralogs (green) data presented in the upper panel of Figure 7A. Significant variation was observed among groups by one-way ANOVA (FP KW statistic = 9.161, P = 0.0272; PIP5K1A KW statistic = 25.48, P <0.0001; PIP5K1B KW statistic = 34.88, P <0.0001; PIP5K1C KW statistic = 55.57, P <0.0001). Significant results are highlighted in bold and n values are displayed.

EGFP	BFP	PIP4K2A	PIP4K2B
PIP4K2A (n=90)	>0.9999		
PIP4K2B (n=90)	**0.0341**	0.1387	
PIP4K2C (n=90)	>0.9999	>0.9999	0.1533
PIP5K1A	+BFP	+ PIP4K2A	+ PIP4K2B
+ PIP4K2A (n=90)	0.0679		
+ PIP4K2B (n=90)	>0.9999	0.9888	
+ PIP4K2C (n=90)	**<0.0001**	0.1445	**0.0016**
PIP5K1B	+BFP	+ PIP4K2A	+ PIP4K2B
+ PIP4K2A (n=90)	**0.0119**		
+ PIP4K2B (n=90)	**<0.0001**	0.3703	
+ PIP4K2C (n=90)	**<0.0001**	0.1917	>0.9999
PIP5K1C	+BFP	+ PIP4K2A	+ PIP4K2B
+ PIP4K2A (n=90)	>0.9999		
+ PIP4K2B (n=90)	**0.0237**	**0.0167**	
+ PIP4K2C (n=90)	**<0.0001**	**<0.0001**	**0.0032**

Journal of Cell Science • Supplementary information

Table S7. P values from Kruskal-Wallis test with Dunn's correction for multiple comparisons for PIP4K paralogs (blue) biosensor data presented in the lower panel of Figure 7A. Significant variation was observed among groups by one-way ANOVA (FP KW statistic = 136.3, P <0.0001; PIP5K1A KW statistic 222.3, P <0.0001; PIP5K1B KW statistic = 236.6, P <0.0001; PIP5K1C KW statistic = 209.4, P <0.0001). Significant results are highlighted in bold and n values are displayed.

EGFP	BFP	PIP4K2A	PIP4K2B
PIP4K2A (n=90)	**<0.0001**		
PIP4K2B (n=90)	**<0.0001**	**<0.0001**	
PIP4K2C (n=90)	**<0.0001**	**<0.0001**	>0.9999
PIP5K1A	+BFP	+ PIP4K2A	+ PIP4K2B
+ PIP4K2A (n=90)	**<0.0001**		
+ PIP4K2B (n=90)	**<0.0001**	>0.9999	
+ PIP4K2C (n=90)	**<0.0001**	**<0.0001**	**<0.0001**
PIP5K1B	+BFP	+ PIP4K2A	+ PIP4K2B
+ PIP4K2A (n=90)	**<0.0001**		
+ PIP4K2B (n=90)	**<0.0001**	**0.0001**	
+ PIP4K2C (n=90)	**<0.0001**	**<0.0001**	0.5067
PIP5K1C	+BFP	+ PIP4K2A	+ PIP4K2B
+ PIP4K2A (n=90)	**<0.0001**		
+ PIP4K2B (n=90)	**<0.0001**	0.193	
+ PIP4K2C (n=90)	**<0.0001**	>0.9999	0.0544

Journal of Cell Science • Supplementary information

Table S8. Plasmids used in this study.

Plasmid	Vector	Insert	Reference	Addgene Plasmid #
EGFP	pEGFP-C1	EGFP	This study	
EGFP-PIP5K1A	pEGFP-C1	EGFP:PIP5K1A	This study	202720
EGFP-PIP5K1A^{D322K}	pEGFP-C1	EGFP:PIP5K1A(D322K)	This study	
EGFP-PIP5K1B	pEGFP-C1	EGFP:PIP5K1B	This study	202722
EGFP-PIP5K1B^{D266K}	pEGFP-C1	EGFP:PIP5K1B(D266K)	This study	202723
EGFP-PIP5K1C	pEGFP-C2	EGFP:PIP5K1C	(Paolo et al., 2002)	
EGFP-PIP5K1C^{D316K}	pEGFP-C2	EGFP:PIP5K1C(D316K)	(Paolo et al., 2002)	
TagBFP2-Mss4-Kina	pTagBFP2-C1	mTagBFP2:*S. cerevisiae* Mss4(377-756)	This study	202724
TagBFP2-Mss4^{D636K}-Kina	pTagBFP2-C1	mTagBFP2:*S. cerevisiae* Mss4(377-756)(D636K)	This study	202725
TagBFP2	pTagBFP2-C1	mTagBFP2	This study	
TagBFP2-PIP4K2A	pTagBFP2-C1	mTagBFP2:PIP4K2A	(Lundquist et al., 2018) This study	202726
TagBFP2-PIP4K2A^{D273K}	pTagBFP2-C1	mTagBFP2:PIP4K2A(D266K)	This study	202727
TagBFP2-PIP4K2B	pTagBFP2-C1	mTagBFP2:PIP4K2B	(Lundquist et al., 2018) This study	202728
TagBFP2- PIP4K2B^{D278K}	pTagBFP2-C1	mTagBFP2:PIP4K2B (D278K)	This study	202729
TagBFP2-PIP4K2C	pTagBFP2-C1	mTagBFP2:PIP4K2C	This study	202730
TagBFP2- PIP4K2C^{D280K}	pTagBFP2-C1	mTagBFP2:PIP4K2C (D280K)	This study	202731
mCherry-FKBP-PIP4K2A	pmCherry-C1	mCherry:FKBP1A(3-108):[GGSA]$_4$GG:PIP4K2A	(Lundquist et al., 2018) This study	202732
mCherry-FKBP-PIP4K2A^{D273K}	pmCherry-C1	mCherry:FKBP1A(3-108):[GGSA]$_4$GG:PIP4K2A (D266K)	This study	202733
mCherry-FKBP-PIP4K2B	pmCherry-C1	mCherry:FKBP1A (3-108):[GGSA]$_4$GG:PIP4K2B	(Lundquist et al., 2018) This study	202734
mCherry-FKBP-PIP4K2B^{D278K}	pmCherry-C1	mCherry:FKBP1A (3-108):[GGSA]$_4$GG:PIP4K2B (D280K)	This study	202735
mCherry-FKBP-PIP4K2C	pmCherry-C1	mCherry:FKBP1A(3-108):[GGSA]$_4$GG:PIP4K2C	This study	202736
mCherry-FKBP-PIP4K2C^{D280K}	pmCherry-C1	mCherry:FKBP1A(3-108):[GGSA]$_4$GG:PIP4K2C (D278K)	This study	202737
EGFP-INPP5E	pEGFP-C2	EGFP:*Mus musculus* INPP5E	(Roberts et al., 2005)	
Lyn$_{11}$-FRB-iRFP	piRFP-N1	LYN(1-11):MTOR(2021-2113):iRFP	(Hammond et al., 2014)	
TagBFP2-FKBP-INPP5E	pTagBFP2-C1	mCherry:FKBP1A(3-108):[GGSA]$_4$GG:INPP5E(214-644)	(Hammond et al., 2014)	
TagBFP2-FKBP-INPP5E^{D477N}	pTagBFP2-C1	mCherry:FKBP1A(3-108):[GGSA]$_4$GG:INPP5E(214-644)(D477N)	This study	
mCherry-Mss4-Kina	pmCherry-C1	mCherry:*S. cerevisiae* Mss4 (377-756)	This study	202739
mCherry-Mss4^{D636K}-Kina	pmCherry-C1	mCherry:*S. cerevisiae* Mss4 (377-756)(D636K)	This study	202740
TagBFP2-FKBP-PIP5K1C_Kina-HD	pTagBFP2-C1	mTagBFP2:FKBP1A(3-108):[GGSA]$_4$GG:PIP5K1C(79-366)(D101R/R304D)	This study	202741
TagBFP2-FKBP-PIP5K1C_Kina-HD^{D316K}	pTagBFP2-C1	mTagBFP2:FKBP1A (3-108) : [GGSA]$_4$GG : PIP5K1C(79-366) (D101R / R304D / D316K)	This study	202742
HAx3-AChR-M3	pcDNA3.1	HAx3:CHRM3(2-590)	J. Wess	

Journal of Cell Science • Supplementary information

TagBFP2-HRAS-CAAX	pTagBFP2-C1	TagBFP2:HRAS(172-189)	(Goulden et al., 2019)	
iRFP713-FRB-PIP5K1A	piRFP-C1	iRFP713:MTOR(2021-2113):GGSA$_2$:PIP5K1A	This study	202743
iRFP713-FRB-Mss4-Kina	piRFP-C1	iRFP713:MTOR(2021-2113):GGSA$_2$:*S. cerevisiae* Mss4 (377-756)	This study	202744
TagBFP2-FKBP-CYB5Atail	pTagBFP2-C1	mTagBFP2:FKBP1A(3-108):[GGSA]$_4$GG:CYB5A(100-134)	(Zewe et al., 2018)	108141
mCherry-FKBP-PIP4K2A-Kina	pmCherry-C1	mCherry:FKBP1A(3-108) : [GGSA]$_4$GG :PIP4K2A (33-406)	This study	202745
EGFP-INPP5E-CAAX	pEGFP-C1	EGFP:INPP5E(214-644):HRAS(172-189)	This study	202746
Lyn$_{11}$^{C3S}-EGFP	pEGFP-N1	LYN (1-11) (C3S):EGFP	This study	202747
EGFP-Lyn$_{11}$^{C32S}-PIP4K2A	pEGFP-C1	EGFP:LYN (1-11) (C3S):PIP4K2A	This study	202748
Tubby$_C$-EGFP	pEGFP-N1	*Mus musculus* Tub (243-505):EGFP	(Quinn et al., 2008)	
Tubby$_C$^{R332H}-EGFP	pEGFP-N1	*Mus musculus* Tub (243-505) (R332H):mCherry	(Quinn et al., 2008)	
Tubby$_C$-mCherry	pmCherry-N1	*Mus musculus* Tub (243-505):mCherry	(Quinn et al., 2008)	
Tubby$_C$^{R332H}-mCherry	pmCherry-N1	*Mus musculus* Tub (243-505) (R332H):EGFP	(Quinn et al., 2008)	
PH-PLCδ1-EGFP	pEGFP-N1	PLCD1v2(1-170):EGFP	(Várnai and Balla, 1998)	
mCherry-P4Mx2	pmCherry-C1	mCherry:L.pneumophila SidM(546-647):SidM(546-647)	(Hammond et al., 2014)	
mCherry-MAPPER	pmCherry-C1	mCherry:MAPPER	(Chang et al., 2013)	
NES-mCherry-PH-ARNO$^{2G-I303Ex2}$	pEGFP-C1	*X. Laevis* map2k1.L(32-44):mCherry:CYTH2(252-399) (I303E):GGSGGVDM : CYTH2(252-399) (I303E)	(Goulden et al., 2019)	
R-GECO1.2	pcDNA3	R-GECO1(M164R / I166V / V174L / F222L / N267S / S270T / I330M / L419I)	(Wu et al., 2013)	
iRFP670-PIP5K1A	iRFP670-C1	iRFP670:PIP5K1A	This study	202749
iRFP670-PIP4K2C	iRFP670-C1	iRFP670:PIP4K2C	This study	202750
PH-PLCδ1-mNGx1	pmNG2-N1	PLCD1v2(1-170):mNG2	This study	202751
PH-PLCδ1-mNGx2	pmNG2-N1	PLCD1v2(1-170): mNG2:mNG2	This study	202752
PH-PLCδ1-mNGx3	pmNG2-N1	PLCD1v2(1-170): mNG2:mNG2:mNG2	This study	202753
His6-OCRL	pFastBac1	His6-MBP-Asn10-TEV-Gly5-OCRL (901isoform) (234-539)	(Hansen et al., 2019)	
His6-PIP4K2A	pETM	His6-SUMO3- Gly5-PIP4K2A (1-406)	This study	
His6-Mss4	pFastBac1	His6-MBP-TEV-Gly5-Mss4 (379-779)	(Hansen et al., 2021)	
His6-PIP5K1A	pFastBac1	His6-MBP-TEV-Gly5-PIP5K1A (1-546)	(Hansen et al., 2021)	
His6-PH-PLCδ1	pETM	His6-SUMO3-Gly5-PLCD1 (11-140)	(Hansen et al., 2019)	

Journal of Cell Science • Supplementary information

Reference for Table S8

Chang, C.-L., Hsieh, T.-S., Yang, T. T., Rothberg, K. G., Azizoglu, B. D., Volk, E., Liao, J.-C. and Liou, J. (2013). Feedback regulation of receptor-induced Ca^{2+} signaling mediated by E-Syt1 and Nir2 at endoplasmic reticulum-plasma membrane junctions. *Cell Rep.* **5**, 813-825. doi:10.1016/j.celrep.2013.09.038

Goulden, B. D., Pacheco, J., Dull, A., Zewe, J. P., Deiters, A. and Hammond, G. R. V. (2019). A high-avidity biosensor reveals plasma membrane PI(3,4)P2 is predominantly a class I PI3K signaling product. J Cell Biol. 218, 1066-1079. doi:10.1083/jcb.201809026

Lundquist, M. R., Goncalves, M. D., Loughran, R. M., Possik, E., Vijayaraghavan, T., Yang, A., Pauli, C., Ravi, A., Verma, A., Yang, Z. et al. (2018). Phosphatidylinositol-5-phosphate 4-kinases regulate cellular lipid metabolism by facilitating autophagy. *Mol. Cell* **70**, 531-544.e9. doi:10.1016/j.molcel.2018.03.037

Hansen, S. D., Lee, A. A. and Groves, J. T. (2021). Membrane-mediated dimerization potentiates PIP5K lipid kinase activity. *Biorxiv* 2021.09.21.461304.

Paolo, G. D., Pellegrini, L., Letinic, K., Cestra, G., Zoncu, R., Voronov, S., Chang, S., Guo, J., Wenk, M. R. and Camilli, P. D. (2002). Recruitment and regulation of phosphatidylinositol phosphate kinase type 1γ by the FERM domain of talin. *Nature* **420**, 85-89. doi:10.1038/nature01147

Roberts, H. F., Clarke, J. H., Letcher, A. J., Irvine, R. F. and Hinchliffe, K. A. (2005). Effects of lipid kinase expression and cellular stimuli on phosphatidylinositol 5–phosphate levels in mammalian cell lines. *FEBS Lett.* **579**, 2868-2872. doi:10.1016/j.febslet.2005.04.027

Várnai, P. and Balla, T. (1998). Visualization of phosphoinositides that bind pleckstrin homology domains: calcium- and agonist-induced dynamic changes and relationship to Myo-[3H]inositol-labeled phosphoinositide pools. *J. Cell Biol.* **143**, 501-510. doi:10.1083/jcb.143.2.501

Wu, J., Liu, L., Matsuda, T., Zhao, Y., Rebane, A., Drobizhev, M., Chang, Y.-F., Araki, S., Arai, Y., March, K. et al. (2013). Improved orange and red Ca^{2+} indicators and photophysical considerations for optogenetic applications. *Acs. Chem. Neurosci.* **4**, 963-972. doi:10.1021/cn400012b

Table S9. HDR and gRNA sequences for PIP4Ks.

Gene	gRNA sequences
PIP4K2A	AGGTAGAAGAGAACTACGTC
PIP4K2B	CAGGAGGTTACGTCAAGATG
PIP4K2C	GGAGACTATGGCGTCCTCCT
	HDR sequences
PIP4K2A	AAGCCACCTCTGTCCATCCAATGTTCATGTCTGTCCGAGGCTGCGCAGGAGGTTACATCATATCGGTAAAGG<br>CCTTTTGCCACTCCTTGAAGTTGAGCTCGGTACCACTTCCTGGACCTTGAAACAAAACTTCCAATCCGCCACC<br>CGTCAAGATGTGGCCAATAAAGTCCAAAAAGCGCTTTGAATACTGTTCTGGGTTC
PIP4K2B	GATCCCCGACCCCATATCCAGCTCTCTGGCTCTGGCTGAAGGTAGAAGAGAACTACATCATATCGGTAAAGG<br>CCTTTTGCCACTCCTTGAAGTTGAGCTCGGTACCACTTCCTGGACCTTGAAACAAAACTTCCAATCCGCCACC<br>CGTCAGGATGTTGGACATAAACTCGTTGAAGCGTTTGGAGTACTGCTCAGGGTTC
PIP4K2C	CCGCTTCCGGGGTCGGGCGCCTGGATAGCTGCCGGCTCCGGCTTCCACTTGGTCGGTTGCGCGGGAGACT<br>ATGACCGAGCTCAACTTCAAGGAGTGGCAAAAGGCCTTTACCGATATGATGGGTGGCGGCATGGCGTCCTC<br>CTCGGTCCCACCAGCCACGGTATCGGCGGCGACAGCAGGCCCCGGCCCAGGTTTCGGCT

Table S10. Genotyping primers for PIP4Ks.

Gene	Primer F	Primer R
PIP4K2A	TGTAAACAAGGAGGTTTGCTTC	TTTGGGGAAATCATAAACACTATG
PIP4K2B	GTCTCATTGCTAAAGCCCTCC	GAACTCAACCTGAATCAGCCTC
PIP4K2C	TCGCCCTGTTCGCGCGTCCG	GGTGGGACCGAGGAGGACGC

Journal of Cell Science • Supplementary information
