# Supplementary material for: A novel homeostatic mechanism tunes PI(4,5)P2-dependent signaling at the plasma membrane
Source: J Cell Sci. 2023 Aug 29;136(16):jcs261494. doi: 10.1242/jcs.261494 (PMC10482388; doi:10.1242/jcs.261494)
Supplement: Supplementary file 1 [file joces-136-261494_review_history.pdf]

A novel homeostatic mechanism tunes PI(4,5)P_2-dependent signaling at the plasma membrane

Rachel C. Wills, Colleen P. Doyle, James P. Zewe, Jonathan Pacheco, Scott D. Hansen, Gerald R. V. Hammond
DOI: 10.1242/jcs.261494

Editor: Michael Way

Review timeline

Submission to Review Commons:	15 July 2022
Submission to Journal of Cell Science:	19 July 2023
Accepted:	19 July 2023

Reviewer 1

Evidence, reproducibility and clarity

In this manuscript, authors address how PIP4K regulates tonic plasma membrane (PM) PI(4,5)P2 levels which are generated by major PI(4,5)P2 synthesis enzyme, PIP5K by using PIP4K and PIP5K overexpressing cells or acutely manipulating PM PI(4,5)P2 levels by the chemically induced dimerization (CID) system. Additionally, authors assessed effect of direct interaction between PIP4K and PIP5K by using supported lipid bilayers (SLBs) and purified PIP4K and 5K. Authors also were successful in monitoring dynamics of endogenous PIP4K by using a split fluorescent protein approach. Through this study, authors propose a model of PI(4,5)P2 homeostatic mechanism that PIP4Ks sense elevated PM PI(4,5)P2 by PIP5Ks, are recruited to the PM, and bind to PIP5Ks to inhibit PIP5Ks activity.

1. Although authors mention methods of statistical analysis in materials and methods, they did not present the results of statistical analysis in the figures. The quantitative data should be presented with statistical analysis data, which is important for showing where convincing differences between treatment groups are found.

2. Fig. 1D. Fig. 1D and Fig. 3A should be presented together because these are exactly same set of cells and information of each PIP4K and PIP5K membrane localization could be important for understanding mechanisms of inhibitory effect of PIP4Ks. Authors claimed that over-expression of all three PIP4K isoforms were able to attenuate the elevated PM PI(4,5)P2 levels caused by PIP5K over-expression. However, in Fig. 3A, PIP4K2A was recruited to PM by both PIP5K1A and PIP5K1C but looks only attenuated PIP5K1A, but not PIP5K1C, overexpression mediated PM PI(4,5)P2 elevation (Fig. 1D). PIP4K2C was less recruited to the PM than PIP4K2A and 2B in PIP5K1A overexpressing cell (Fig. 3A) but PIP4K2A, B and C isoforms equally attenuated increase of PM PI(4,5)P2 in PIP5K1A overexpressing cell (Fig. 1D). It is likely that efficiency of inhibitory effect of each PIP4K isoform is different by co-overexpressed PIP5K isoform. These images should be more carefully documented with Fig. 1D and Fig. 3A together.

3. Fig. 1F. It seems that PIP4K2A accelerated PIP5K, but not Mss4, dependent PI(4,5)P2 generation before PI(4,5)P2 reaches 28,000 lipids/um2. Is this significant? If so, why did this happen?

4. Fig. 3B. In this figure, authors only presented images after Rapa treatment. Therefore, it is not clear what these results mean. Before Rapa treatment, where did bait proteins and NG2- PIP4K2C localize? If ePIP4K2C delta PM intensity (ER:PM/PM) increase, does that mean increase in ER:PM intensity or decrease in PM intensity? According to Figure legend, PI(4,5)P2 indicator TubbycR332H was co-transfected, but those images are not shown in the figure. Images of PI(4,5)P2 indicator

© 2023. Published by The Company of Biologists under the terms of the Creative Commons Attribution License (https://creativecommons.org/licenses/by/4.0/).

also should be presented to show whether after Rapa treatment PI(4,5)P2 increased at ER-PM contact sites, because that could be critical for the conclusion that "The use of Mss4 ruled out an effect of enhanced PI(4,5)P2 generation at contact sites, since this enzyme increases PI(4,5)P2 as potently as PIP5K1A (Fig. 1A), yet does not cause recruitment of PIP4K2C". Is this conclusion consistent with Fig. 2F and G?

5. Fig. 3C and D. Based on results of Fig. 3C and D, authors concluded that "PIP4K2C binding to PI(4,5)P2-containing SLBs was greatly enhanced by addition of PIP5K to the membranes, but not Mss4". I don't think Fig. 3C and D are comparable because experimental conditions are different. While lipid composition of SLB used in Fig. 3C was 2% PI(4,5)P2, 98% DOPC, in Fig. 3D, it was 4% PI(4,5)P2, 96% DOPC. And also, in Fig. 3C, PIP5K1A was added to SLB at the time about 50 sec, whereas in Fig. 3D, Mss4 was added at 600 sec. It seems that in Fig. 3D, PIP4K2A was already saturated on SLB before adding Mss4. These two experiments must be performed under the same conditions.

6. Overall results discussed in the text are very compressed referring readers to the 4 multi- panel complex figures with elaborate figure legends. While it is possible to figure out what the authors' studies and results are, it is quite a laborious process.

Minor comments:
1. Fig. 2D. The purified 5-phosphatase used in Fig. 2D is INPP5E but described in figure legend and materials and methods ass OCRL. Which one is correct?
2. Fig. 3B. Indicate which trace represents PIP5K1A, Lyn11 or Mss4.
3. Fig. 4C. X-axis label. Is "Time (min)" correct? Or should it be "Time (sec)".

Significance

The finding that PIP4K itself is a low-affinity PI(4,5)P2 binding protein and sense increases of PM PI(4,5)P2 generated by PIP5K to control tonic PI(4,5)P2 levels by inhibiting PIP5K activity is a novel concept. However, inhibition of PIP5K by PIP4K and importance of the inhibitory effect of PIP4K in PI3K signaling pathway have previously been reported (ref 24). This reduces the novelty of the current work somewhat however, the authors do provide evidence for dual interactions of PIP4K (PIP2, PIP5K), which the previous report did not.

The reviewers have expertise in PLC- and PIP5K-related signaling pathways.

<u>Reviewer 2</u>

Evidence, reproducibility and clarity

Summary:
This paper proposes that the enzyme PIP4K2C is a negative regulator of the synthesis of PI(4,5)P2 and that it does so by dampening the activity of PIP5K which is the enzymatic activity responsible for producing the major pool of PI(4,5)P2 in cells.

Significance

Nature and Significance of the advance:

Although the findings of the paper are presented as a major new advance, the observation that PIP4K might acts as a negative regulator of PIP2 synthesis has been previously presented in two previous publications. The significance of this paper is that it also shows the same point in another model system.

PIP4Ks Suppress Insulin Signaling through a Catalytic-Independent Mechanism Diana G Wang 1, Marcia N Paddock 2, Mark R Lundquist 3, Janet Y Sun 3, Oksana Mashadova 3, Solomon Amadiume

© 2023. Published by The Company of Biologists under the terms of the Creative Commons Attribution License (https://creativecommons.org/licenses/by/4.0/).

3, Timothy W Bumpus 4, Cindy Hodakoski 3, Benjamin D Hopkins 3, Matthew Fine 3, Amanda Hill 3, T Jonathan Yang 5, Jeremy M Baskin 4, Lukas E Dow 6, Lewis C Cantley 7

PMID: 31091439; PMCID: PMC6619495;DOI: 10.1016/j.celrep.2019.04.070 and Phosphatidylinositol 5 Phosphate 4-Kinase Regulates Plasma-Membrane PIP3 Turnover and Insulin Signaling.

Sharma S, Mathre S, Ramya V, Shinde D, Raghu P.Cell Rep. 2019 May 14;27(7):1979-1990.e7. doi: 10.1016/j.celrep.2019.04.084.PMID: 31091438

Both of these studies show that in cells lacking PIP4K, during signalling the levels of PIP2 rise much greater than in wild type cells. Indeed the Cantley lab paper (Wang et.al) have shown that this is likely due to an increase in PIP5K activity, using an in vitro assay. They have further disrupted the interaction between PIP4K and PIP5K and demonstrated the importance of this interaction in the enhanced levels of PIP2.

Likewise although the authors have claimed that no mechanisms have claimed that there are no mechanisms reported to sense and downregulate PIP2 resynthesis. It is suggested that they read and consider the following recent paper which studies Pip2 resynthesis during GPCR triggered PLC signalling.

Kumari A, Ghosh A, Kolay S and Raghu P*. Septins tune lipid kinase activity and PI(4,5)P 2 turnover during G-protein-coupled PLC signalling in vivo. Life Sci Alliance. 2022 Mar 11;5(6):e202101293. doi: 10.26508/lsa.202101293. Print 2022 Jun.

Likewise there are other earlier papers in the literature which have studied possible PIP2 binding proteins as sensors for this lipid.

Technical standards: The work is done to a high technical standard.

Major and Minor Comments

Comments for Figure 1
1. Does catalytically dead isoform of PIP4K2B and 2C also yield the same result as a catalytically dead version of PIP4K2A in Fig 1B?

2. The labelling on y-axis for PI(4,5)P2 biosensor intensity ratio is PM/cell at some places, PM/Cyt or PM/Cyto in some places. It is recommended to make it uniform across all the panels.

Comments for Figure 2
1. The claim that PI(4,5)P2 production is sufficient to recruit PIP4K2C to the PM can be ascertained further if one is able to do an experiment where PI(4,5)P2 is ectopically expressed in some compartment of the cell which is non-native to PI(4,5)P2 and as a consequence of this PIP4K2C is recruited to this non-native compartment.

2. In the entire figure 2, to establish that PI(4,5)P2 is necessary and sufficient for PM localisation of PIP4K, PIP4K2C is used as the PIP4K isoform on the basis that it is highly abundant in HEK293 cells. But PIP4K2A is localised mainly at the plasma membrane and here we are discussing about PI(4,5)P2 regulation at the PM . Can experiments be done with isoforms 2A and 2B as well? Can acute depletion of PI(4,5)P2 lead to the membrane dissociation of the isoform 2A as well? This will help us in understanding if there is an isoform specific difference in sensing PI(4,5)P2 levels which will help us in targeting specific isoform as therapeutic targets.

3. In Figure 1A, it is shown that overexpression of a catalytically dead PIP5K 1A/1B/1C is still able to increase PI(4,5)P2 levels. In the figure 2E, expression of homodimeric mutant of PIP5K domain which is a way to increase catalytic activity of PIP5K, increases PI(4,5)P2 levels which is consistent with the inferences from Fig, 1 , but what is surprising is a catalytically dead variant not being able to do so. Why is there a discrepancy between Fig. 1A and Fig. 2E? If the homodimeric mutant is the reason, then it is not clear in the explanation.

4. Show the loading control in Fig 2A western.

© 2023. Published by The Company of Biologists under the terms of the Creative Commons Attribution License (https://creativecommons.org/licenses/by/4.0/).

5. In the figure 2D, in the legend OCRL is written. So, the labelling in the panel should also be changed to OCRL from INPP5E. It is intermixed.

6. In the figure 2E, can the labelling be changed from HD to something more self-explanatory for homodimeric mutant of PIP5K domain?

7. In Fig. 2E, PIP5K expression is acute and in Fig. 2F Mss4 expression is chronic, both of which is able to recruit PIP4K2C to the plasma membrane. How can a likewise argument be drawn out of these two experiments when one is acute and the other one is a chronic expression? It is suggested to do an FRB-FKBP experiment for Mss4 as well.

8. In the text, Fig. 2G and 2H is written for PIP4K2C, but in the corresponding panels and legends, it is an assay for purified PIP4K2A on SLBs. Kindly resolve the discrepancy.

Comments for Figure 3

1. Kindly explain a bit in detail why the baits were now targeted to ER-PM contact sites. It is not self-explanatory.

2. The conclusions for Fig. 3 most likely hints towards the possibility of PIP4K and PIP5K interaction being independent of PI(4,5)P2 levels. Well, Fig. 3C and 3D does suggest a direct interaction, but can other protein-protein interaction assays be used to establish the direct interaction of PIP4K with PIP5K such as FRET or Yeast two hybrid as assays scoring for interaction?

3. Conceptually a direct interaction can be explained to some extent from Fig. 3 but extending it to be an inhibitory interaction is not right without a direct experiment. Can an experiment be done with PI4P enriched SLB, wherein you put just PIP5K purified protein vs PIP5K+PIP4K combination and measure the % mol of PI(4,5)P2 produced using a probe. That will be suggestive of a negative interaction.

4. In Figure 3B, the FRB tagged constructs are magenta coded and PIP4K2C is cyan. Kindly change the labelling of the FRB constructs on the y axis to magenta so that it goes with what is written in the legend. It will also be appreciated to show a colocalization quantification between the magenta (FRB constructs) and cyan (PIP4K2C) post rapamycin addition and not just the intensity for ER-PM recruited PIP4K2C.

5. Again, in the text , the description is written for PIP4K2C but in the result panel and legend (Fig. 3C and Fig. 3D), PIP4K2A is mentioned. Kindly resolve the discrepancy

Comments for Figure 4

1. In the Fig. 4B, it will be appreciated to show statistical significance in terms of R2 value for commenting on the linear response.

Comments for Discussion

1. Discussion can be in general a bit more detailed which is suggestive of future experiments to do that can shed more light on the interaction such as which residues in PIP4K interacts with PIP5K to negatively regulate it.

2. In the discussion, more light can be shed on the fact that Mss4 in spite of being a 5- kinase is not negatively regulated by PIP4K and the fact that PIP4K is present only in metazoans suggests that this fine tuning of PI(4,5)P2 levels is specific to metazoans. Another insight could be in the direction, that Fig 4. tells PI3K, but not calcium signaling is modulated by this fine tuning and interestingly class I PI3K is also an enzyme specific to metazoans. Hence, unlike yeast, metazoans rely on growth factor signalling processes, hence regulation of PI(4,5)P2 by PIP4K and hence Class I PI3K and PI(3,4,5)P3 could be a process relevant to metazoans.

© 2023. Published by The Company of Biologists under the terms of the Creative Commons Attribution License (https://creativecommons.org/licenses/by/4.0/).

Audience: cell biologists and biochemists interested in PIp2 signalling and PIP kinases

My expertise: PIP2 and PIP kinases

Reviewer 3

Evidence, reproducibility and clarity

Using state of the art imaging techniques the authors try to address how cells sense PI(4,5)P2 levels and regulate PIP5Ks to maintain an optimal level since any dysregulation of PI(4,5)P2 levels can have significant effects on the functioning of the cell and led to numerous disease states, such as cancers.

The key conclusions are convincing and importantly validate previous disputed findings made by Wang et al. (Cell Reports 2019) using different and more rigorous methods, however unfortunately due to the Wang et al publication the overall novelty of this study is lacking. A suggestion to the authors is to state/explain with text more clearly how their findings are more precise and higher quality than the previous report and why their findings are necessary and significant to drive the field forward.

Further, experiments in the study were performed in vitro in cultured cells using overexpression methods making the physiological significance a bit unclear and the enthusiasm of the main discovery dampened. With that being said these findings are worthy of publication in order to advance the field and understanding of how the PIP kinase families are regulated and maintain PIP2 homeostasis which is important for life.

Minor and easily addressable experiments should be performed by the authors the following. Further, many of these experimental issues can easily go in supplemental materials

1. Include western blots for the constructs to compare expression levels.

2. For Figure 1A, what is the source of the observed increase in PI(4,5)P2, how do the authors take into account the role of endogenous PIP5Ks?

3. For Figure 1B, could the authors comment on the intracellular distribution of PI(4,5)P2. How are they able to reliably distinguish their signal between plasma membrane and intracellular localizations and conclude that PIP2 on the plasma membrane is decreased?

4. Please include statistics for all image- based quantitation analysis.

5. Could the authors comment on the ability of PIP4K to have affinity for its own product? How does PIP4K sense membrane PI(4,5)P2 since these kinases are mostly cytoplasmic?

6. Do the authors have any other experiments to substantiate the binding of the two PIP kinases, similar to the Wang et al findings? Is the N-term motif required? Is it possible to disrupt that interaction and show the phenotype?

7. With the overexpression studies in Figure 1, do the authors see any changes in signaling when they just overexpress PIP5Ks versus in combination with PIP4Ks to show that the changes in plasma membrane PI(4,5)P2 can affect downstream signaling?

Significance

Overall, as mentioned above because of the 2019 Wang et al report the novelty is diminished, however using completely alternate methods and sophisticated microscopy this body of work indeed advances the field and provides further believable evidence of the PIP kinase families communicating in higher organisms which is required to maintain PIP2 levels shedding light on many of the findings that were previously unexplained surrounding the PIP4K studies. Further, the

© 2023. Published by The Company of Biologists under the terms of the Creative Commons Attribution License (https://creativecommons.org/licenses/by/4.0/).

use of biosensors to describe these findings are new and will enable others in the field to begin to use such tools to investigate potential crosstalk between other lipid kinases.

Author response to reviewers' comments

1. General Statements

Our manuscript details a novel homeostatic feedback loop for the master plasma membrane regulatory molecule, PI(4,5)P2. In this loop, the PIP4K family of PI(4,5)P2-synthesizing enzymes act in a novel, non-enzymatic capacity: they sense PI(4,5)P2 levels and directly inhibit the lipid's synthesis by inhibiting the major enzyme involved in the terminal step of synthesis, PIP5K. The three reviewers seem largely convinced of our data, and provided detailed, insightful and plausible suggestions for revision, which we have now comprehensively provided. This includes substantial new experimental work, including the generation of genomically tagged cell lines to localize all endogenous PIP4K isoforms.

However, all three reviewers questioned the paper's novelty and significance based on recent studies in the literature demonstrating PIP5K inhibition by PIP4Ks [refs 25 & 53 in the manuscript]. We feel that this is an *inaccurate and somewhat unfair assessment of our findings*, since it does not consider our central (and completely unprecedented) finding that PIP4Ks directly sense PI(4,5)P2 levels through low-affinity binding. As well as being a novel finding, this places the previously observed inhibition of PIP5K by PIP4Ks into a completely new paradigm consisting of a complete, enclosed homeostatic feedback loop. This was not demonstrated previously in the literature.

In short, the ***previous findings*** [refs 25 & 53] demonstrated the following:

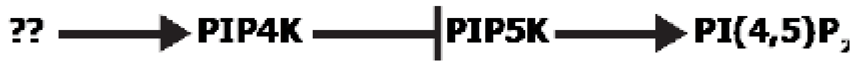

Our work puts the inhibition in the context of a feedback loop:

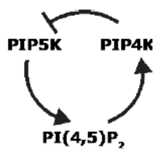

Of course, the reviewers' convergent opinions almost certainly reflect a deficit in our articulation of the novel findings in the original manuscript. We have therefore revised the current version to more clearly emphasize our novel findings.

2. Point-by-point description of the revisions

Reviewer #1

Summary: *In this manuscript, authors address how PIP4K regulates tonic plasma membrane (PM) PI(4,5)P2 levels which are generated by major PI(4,5)P2 synthesis enzyme, PIP5K by using PIP4K and PIP5K overexpressing cells or acutely manipulating PM PI(4,5)P2 levels by the chemically induced dimerization (CID) system. Additionally, authors assessed effect of direct interaction between PIP4K and PIP5K by using supported lipid bilayers (SLBs) and purified PIP4K and 5K. Authors also were successful in monitoring dynamics of endogenous PIP4K by using a split fluorescent protein approach. Through this study, authors propose a model of PI(4,5)P2 homeostatic mechanism that PIP4Ks sense elevated PM PI(4,5)P2 by PIP5Ks, are recruited to the PM, and bind to PIP5Ks to inhibit PIP5Ks activity.*

# 1.1: ***Although authors mention methods of statistical analysis in materials and methods, they did not present the results of statistical analysis in the figures. The quantitative data should be presented with statistical analysis data, which is***

© 2023. Published by The Company of Biologists under the terms of the Creative Commons Attribution License (https://creativecommons.org/licenses/by/4.0/).

important for showing where convincing differences between treatment groups are found.

We agree that statistics are important to fully interpret the data; we have now included the results of statistical tests (non-parametric statistics were used, as the data are not normally distributed) with correction for multiple comparisons. Significant changes are denoted using asterisk notation in **figs. 1A-C, 2B, 5B & 7A**. The full results are now reported as tables:

Fig 1A = table 1; **Fig 1B** = table 2; **Fig. 1C** = table 3; **Fig. 2B** = table 4; **Fig. 5B** = table 5; **Fig 7A** = tables 6 & 7.

***#1.2a**: Fig. 1D. Fig. 1D and Fig. 3A should be presented together because these are exactly same set of cells and information of each PIP4K and PIP5K membrane localization could be important for understanding mechanisms of inhibitory effect of PIP4Ks.*

We struggled when writing the manuscript to reconcile these data into a single figure. The manuscript flows from showing inhibition of PIP5Ks by PIP4Ks in living cells (figs. 1 & 2), then showing low affinity PI(4,5)P2 binding by endogenous PIP4Ks (figs. 3-6) and finally to a direct interaction between PIP4K and PIP5K (fig. 7). We therefore felt that reconciling the data showing attenuated PI(4,5)P2 synthesis with the interaction between PIP4Ks and PIP5Ks, despite being demonstrated in the same experiments, would disrupt the flow of the paper. We therefore request to leave the data in Figs. 2B and 7A, whilst remaining explicit that the data derive from a single experiment.

***#1.2b**: Authors claimed that over-expression of all three PIP4K isoforms were able to attenuate the elevated PM PI(4,5)P2 levels caused by PIP5K over-expression. However, in Fig. 3A, PIP4K2A was recruited to PM by both PIP5K1A and PIP5K1C but looks only attenuated PIP5K1A, but not PIP5K1C, overexpression mediated PM PI(4,5)P2 elevation (Fig. 1D). PIP4K2C was less recruited to the PM than PIP4K2A and 2B in PIP5K1A overexpressing cell (Fig. 3A) but PIP4K2A, B and C isoforms equally attenuated increase of PM PI(4,5)P2 in PIP5K1A overexpressing cell (Fig. 1D). It is likely that efficiency of inhibitory effect of each PIP4K isoform is different by co-overexpressed PIP5K isoform. These images should be more carefully documented with Fig. 1D and Fig. 3A together.*

As the reviewer suggests, we have now expanded our description of these data in both results and discussion; firstly, for the attenuating effects on PI(4,5)P2 synthesis, we write on the **3rd paragraph of p4:** *"We also reasoned that co-expression of PIP4K paralogs with PIP5K might attenuate the elevated PI(4,5)P2 levels induced by the latter. Broadly speaking, this was true, but with some curious paralog selectivity (fig. 2B, statistics reported in table 4): PIP4K2A and PIP4K2B both attenuated PI(4,5)P2 elevated by PIP5K1A and B, but not (or much less so) PIP5K1C; PIP4K2C, on the other hand, attenuated PIP5K1A and was the only paralog to significantly attenuate PIP5K1C's effect, yet it did not attenuate PIP5K1B at all."*

On the relative ability of PIP5Ks to localize PIP4Ks we focus on the key result, writing on the **2nd paragraph of p7:** *"When co-expressing EGFP-tagged PIP5Ks and TagBFP2-tagged PIP4K2s, we found that PIP5K paralogs' PM binding is largely unaffected by PIP4K over-expression (**fig. 7A**, upper panel and **table 6**), whereas all three paralogs of PIP4K are strongly recruited to the PM by co-expression of any PIP5K (**fig. 7A**, lower panel and **table 7**)…"*

And finally, we describe a more nuanced discussion of the possible implications for differential inhibition of PIP5K isoforms by PIP4Ks in the discussion, starting in **the first paragraph on p. 11**: *"Despite minor differences in the ability of over-expressed PIP5K paralogs to recruit over-expressed PIP4K enzymes (**fig. 7A**), we observed major differences in the ability of PIP4K paralogs to inhibit PI(4,5)P2 synthesis when over-expressed alone (**fig. 1C**) or in combination with PIP5K (**fig. 2B**). It is unclear what drives the partially overlapping inhibitory activity, where each PIP5K paralog can be attenuated by 2 or 3 PIP4Ks. This is however reminiscent of the biology of the PIPKs, where there is a high degree of redundancy among them, with few unique physiological functions*

© 2023. Published by The Company of Biologists under the terms of the Creative Commons Attribution License (https://creativecommons.org/licenses/by/4.0/).

*assigned to specific paralogs [49]. There may be hints of paralog- specific functions in our data; for example, enhanced PI(4,5)P2 induced by over-expressed PIP5K1C is only really attenuated by PIP4K2C (**fig. 2B**). This could imply a requirement for PIP4K2C in regulating PI(4,5)P2 levels during PLC-mediated signaling, given the unique requirements for PIP5K1C in this process [50,51]. Regardless, a full understanding of paralog selectivity will need to be driven by a detailed structural analysis of the interaction between PIP4Ks and PIP5Ks - which is not immediately apparent from their known crystal structures, especially since PIP4Ks and PIP5Ks employ separate and distinct dimerization interfaces [49].”*

***#1.3**: Fig. 1F. It seems that PIP4K2A accelerated PIP5K, but not Mss4, dependent PI(4,5)P2 generation before PI(4,5)P2 reaches 28,000 lipids/um2. Is this significant? If so, why did this happen?*

We have answered this question with a sentence added to the **1st paragraph on p 8:** *“The ability of PIP4K to bind to PIP5K on a PI(4,5)P2-containing bilayer also potentially explains the slightly accelerated initial rate of PI(4,5)P2 synthesis exhibited by PIP5K1A that we reported in **fig. 2C**, since PIP4K may initially introduce some avidity to the membrane interaction by PIP5K, before PI(4,5)P2 reaches a sufficient concentration that PIP4K-mediated inhibition is effective.”*

***#1.4**: Fig. 3B. In this figure, authors only presented images after Rapa treatment. Therefore, it is not clear what these results mean. Before Rapa treatment, where did bait proteins and NG2-PIP4K2C localize? If ePIP4K2C delta PM intensity (ER:PM/PM) increase, does that mean increase in ER:PM intensity or decrease in PM intensity? According to Figure legend, PI(4,5)P2 indicator TubbycR332H was co-transfected, but those images are not shown in the figure. Images of PI(4,5)P2 indicator also should be presented to show whether after Rapa treatment PI(4,5)P2 increased at ER-PM contact sites, because that could be critical for the conclusion that "The use of Mss4 ruled out an effect of enhanced PI(4,5)P2 generation at contact sites, since this enzyme increases PI(4,5)P2 as potently as PIP5K1A (Fig. 1A), yet does not cause recruitment of PIP4K2C". Is this conclusion consistent with Fig. 2F and G?*

These data now appear in **Fig. 7B**. We have added images showing the pre-rapamycin state to the revised figure. The reference to tubbyc^{R332H} co-expression was an error. In fact, the cells expressed the ER:PM contact site marker MAPPER, which allowed us to quantify ER:PM contact site localization before and after rapamycin induced capture of the baits at these sites. The revised figure appears as follows:

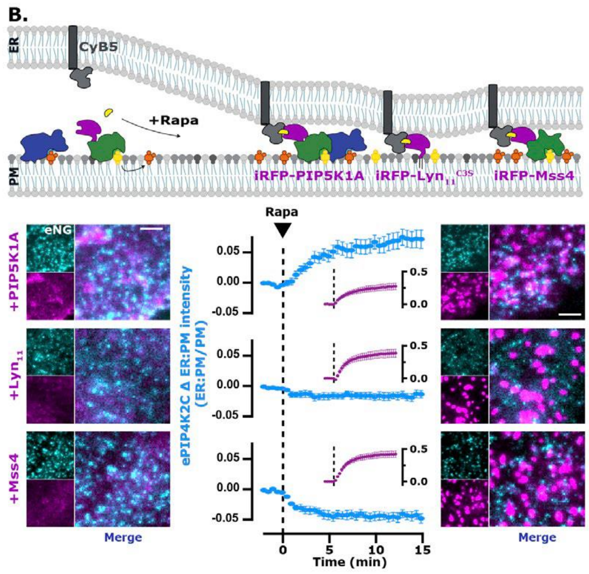

© 2023. Published by The Company of Biologists under the terms of the Creative Commons Attribution License (https://creativecommons.org/licenses/by/4.0/).

(**B**) PIP4K2C interacts with PIP5K1A. Cartoon schematic show the CID system for the generation of ER-PM contact sites between ER- anchored FKBP-CyB5 and PM-anchored FRB-tagged constructs. NG2-PIP4K2C (cyan) cells were transfected with FKBP-CyB5, mCherry-MAPPER and the indicated FRB-tagged construct (magenta). During time-lapse TIRF microscopy, cells were stimulated with 1μM rapa. TIRF images are representative and color-coded to represent fluorescence intensity, as indicated, scale bar is 2.5 μm. NG2-PIP4K2C traces represent mean fluorescence intensities (ER:PM/PM) ± s.e. of 32-39 cells imaged across a minimum of three independent experiments.

The failure of Mss4 to recruit endogenous PIP4K2C is entirely consistent with the old Fig. 2F and G (now 5A and C), since these show PIP4K interaction with PI(4,5)P2 containing lipid bilayers (in Fig. 5C, the PI(4,5)P2 was synthesized by Mss4). We demonstrated that Mss4 is unable to interact with PIP4K2A in **Fig. 7D**.

***#1.5**: Fig. 3C and D. Based on results of Fig. 3C and D, authors concluded that "PIP4K2C binding to PI(4,5)P2-containing SLBs was greatly enhanced by addition of PIP5K to the membranes, but not Mss4". I don't think Fig. 3C and D are comparable because experimental conditions are different. While lipid composition of SLB used in Fig. 3C was 2% PI(4,5)P2, 98% DOPC, in Fig. 3D, it was 4% PI(4,5)P2, 96% DOPC. And also, in Fig. 3C, PIP5K1A was added to SLB at the time about 50 sec, whereas in Fig. 3D, Mss4 was added at 600 sec. It seems that in Fig. 3D, PIP4K2A was already saturated on SLB before adding Mss4. These two experiments must be performed under the same conditions.*

We have repeated these experiments (which now appear in **Fig. 7C & D**) under identical conditions, with the same result.

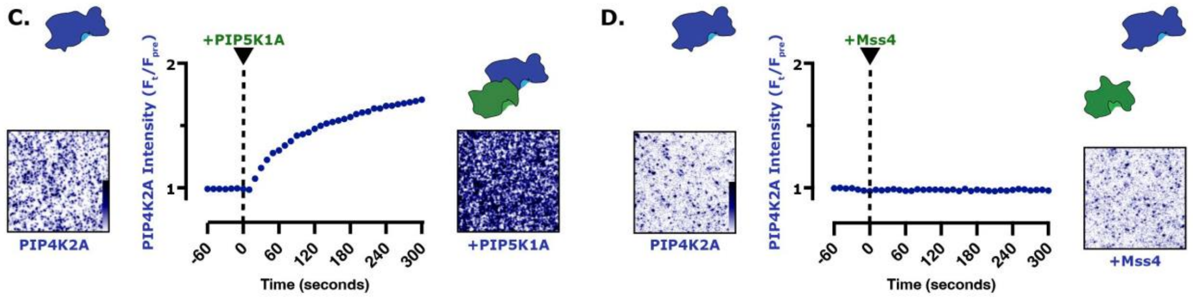

(**C**) Dynamic PIP5K1A dependent membrane recruitment of PIP4K2A to SLBs. TIRF microscopy images show the membrane localization of PIP4K2A in the absence and presence of PIP5K1A. In the absence of PIP5K, 50 nM PIP4K2A displays a low level of membrane recruitment. The addition of 10 nM PIP5K1A, stimulates an immediate and steady increase in PIP4K2A membrane localization. Membrane composition: 2% PI(4,5)P2, 98% DOPC. TIRF images are representative and color-coded to represent fluorescence intensity, as indicated, scale bar is 5 μm. (**D**) Membrane binding of PIP4K2A is insensitive to yeast Mss4 membrane localization. TIRF microscopy images show the membrane localization of PIP4K2A in the absence and presence of Mss4. Following membrane equilibration of 50 nM PIP4K2A, 10 nM Mss4 was added to the imaging chamber. No appreciable change in PIP4K2A localization was observed during membrane absorption of Mss4. Membrane composition: 2% PI(4,5)P2 and 98% DOPC. TIRF images are representative and color-coded to represent fluorescence intensity, as indicated, scale bar is 5 μm.

***#1.6**: Overall results discussed in the text are very compressed referring readers to the 4 multi- panel complex figures with elaborate figure legends. While it is possible to figure out what the authors' studies and results are, it is quite a laborious process.*

We have revised the manuscript to be less compressed and easier to read, with the data now organized as eight figures and the results section split into four sub-sections.

© 2023. Published by The Company of Biologists under the terms of the Creative Commons Attribution License (https://creativecommons.org/licenses/by/4.0/).

Minor comments:

#1.7: *Fig. 2D. The purified 5-phosphatase used in Fig. 2D is INPP5E but described in figure legend and materials and methods ass OCRL. Which one is correct?*

Purified OCRL was indeed used in the supported lipid bilayer experiments. The figure (now **Fig. 4A**) and legend have been corrected – thank you for spotting the error.

#1.8: *Fig. 3B. Indicate which trace represents PIP5K1A, Lyn11 or Mss4.*

The data now appears in **Fig. 7B**, with the traces separated into separate graphs for greater clarity (see response to **#1.4**).

#1.9: *Fig. 4C. X-axis label. Is "Time (min)" correct? Or should it be "Time (sec)".*

Thank you for spotting this typo. It should have indeed been seconds, and this is corrected in the new **fig. 8C.**

Reviewer #1 (Significance (Required)): *The finding that PIP4K itself is a low-affinity PI(4,5)P2 binding protein and sense increases of PM PI(4,5)P2 generated by PIP5K to control tonic PI(4,5)P2 levels by inhibiting PIP5K activity is a novel concept. However, inhibition of PIP5K by PIP4K and importance of the inhibitory effect of PIP4K in PI3K signaling pathway have previously been reported (ref 24). This reduces the novelty of the current work somewhat however, the authors do provide evidence for dual interactions of PIP4K (PIP2, PIP5K), which the previous report did not.*

We appreciate the reviewer's insightful comments and overall appreciation of our work. We agree that previous studies did not detect the dual interaction of PIP4Ks with PIP5Ks and PI(4,5)P2; as we argue strongly in the general comments, we think this actually fits as a complete, enclosed homeostatic feedback loop – which is a significant and novel finding.

Reviewer #2

Summary: *This paper proposes that the enzyme PIP4K2C is a negative regulator of the synthesis of PI(4,5)P2 and that it does so by dampening the activity of PIP5K which is the enzymatic activity responsible for producing the major pool of PI(4,5)P2 in cells.*

Reviewer #2 (Significance (Required)): *Although the findings of the paper are presented as a major new advance, the observation that PIP4K might acts as a negative regulator of PIP2 synthesis has been previously presented in two previous publications. The significance of this paper is that it also shows the same point in another model system.*

PIP4Ks Suppress Insulin Signaling through a Catalytic-Independent Mechanism

Diana G Wang 1, Marcia N Paddock 2, Mark R Lundquist 3, Janet Y Sun 3, Oksana Mashadova 3, Solomon Amadiume 3, Timothy W Bumpus 4, Cindy Hodakoski 3, Benjamin D Hopkins 3, Matthew Fine 3, Amanda Hill 3, T Jonathan Yang 5, Jeremy M Baskin 4, Lukas E Dow 6, Lewis C Cantley 7

PMID: 31091439; PMCID: PMC6619495;DOI: 10.1016/j.celrep.2019.04.070 and Phosphatidylinositol 5 Phosphate 4-Kinase Regulates Plasma-Membrane PIP3 Turnover and Insulin Signaling.

Sharma S, Mathre S, Ramya V, Shinde D, Raghu P.Cell Rep. 2019 May 14;27(7):1979-1990.e7. doi: 10.1016/j.celrep.2019.04.084.PMID: 31091438

Both of these studies show that in cells lacking PIP4K, during signalling the levels of PIP2 rise much greater than in wild type cells. Indeed the Cantley lab paper (Wang

© 2023. Published by The Company of Biologists under the terms of the Creative Commons Attribution License (https://creativecommons.org/licenses/by/4.0/).

et.al) have shown that this is likely due to an increase in PIP5K activity, using an in vitro assay. They have further disrupted the interaction between PIP4K and PIP5K and demonstrated the importance of this interaction in the enhanced levels of PIP2.

Respectfully, we disagree with this assessment, because we believe it doesn't consider the novel, central findings we report: that PIP4Ks sense PI(4,5)P2 levels through direct interaction with the lipid, and that this is what facilitates PIP5K inhibition. These findings were not reported in the prior studies. Nonetheless, the studies are foundational for ours and were cited in our original manuscript (and are still, as refs 25 and 53).

#2.1: Likewise although the authors have claimed that no mechanisms have claimed that there are no mechanisms reported to sense and downregulate PIP2 resynthesis. It is suggested that they read and consider the following recent paper which studies Pip2 resynthesis during GPCR triggered PLC signalling.

Kumari A, Ghosh A, Kolay S and Raghu P. Septins tune lipid kinase activity and PI(4,5)P 2 turnover during G-protein-coupled PLC signalling in vivo. Life Sci Alliance. 2022 Mar 11;5(6):e202101293. doi: 10.26508/lsa.202101293. Print 2022 Jun.*

We have now included a full discussion of this paper in the discussion starting on the **last paragraph of p 9:** *"Since this paper was initially submitted for publication, another study has reported a similar homeostatic feedback loop in Drosophila photoreceptors, utilizing the fly homologue of septin 7 as the receptor and control center [38]. This conclusion is based on the observation that cells with reduced septin 7 levels have enhanced PIP5K activity in lysates, and exhibit more rapid PI(4,5)P2 resynthesis after PLC activation. However, changes in septin 7 membrane localization in response to acute alterations in PI(4,5)P2 levels, as well as direct interactions between PIP5K and septin 7, have yet to be demonstrated. Nevertheless, septin 7 has distinct properties as a potential homeostatic mediator; as a foundational member of the septin family, it is essential for generating all major types of septin filament [39]. Therefore, a null allele for this subunit is expected to reduce the prevalence of the septin cytoskeleton by half. Given that septin subunits are found in mammalian cells at high copy number, around ~10^6 each [29], and the fact that septins bind PI4P and PI(4,5)P2 [40,41], it is likely that septin filaments sequester a significant fraction of the PM PI4P and PI(4,5)P2 through high-avidity interactions. In addition, membrane-bound septins appear to be effective diffusion barriers to PI(4,5)P2 and other lipids [42]. We therefore speculate that septins may play a unique role in systems such as the fly photoreceptor with extremely high levels of PLC-mediated PI(4,5)P2 turnover: The septin cytoskeleton can act as a significant buffer for PI4P and PI(4,5)P2 in such systems, as well as corralling pools of the lipids for use at the rhabdomeres were the high rate of turnover occurs. This is in contrast to the role played by the PIP4Ks, where PI(4,5)P2 levels are held in a narrow range under conditions of more limited turnover, as found in most cells."*

#2.2: Likewise there are other earlier papers in the literature which have studied possible PIP2 binding proteins as sensors for this lipid.

We are only aware of a single, specific example of a similar negative feedback, which is discussed in the **3rd paragraph of p 10:** *"Curiously, although phosphatidylinositol phosphate kinases are found throughout eukarya, PIP4Ks are limited to holozoa (animals and closely related unicellular organisms) [47]. Indeed, we found the PIP5K from the fission yeast, Saccharomyces cerevisiae, does not interact with human PIP4Ks (**fig. 7**) and cannot modulate PI(4,5)P2 levels in human cells without its catalytic activity (**fig. 1**). This begs the question: how do S. cerevisiae regulate their own PI(4,5)P2 levels? Intriguingly, they seem to have a paralogous homeostatic mechanism: the dual PH domain containing protein Opy1 serves as receptor and control center [48], in an analogous role to PIP4K. Since there is no mammalian homolog of Opy1, this homeostatic mechanism appears to have appeared at least twice through convergent evolution. Combined with hints of a role for septins in maintaining PI(4,5)P2 levels [38], the possibility arises that there may yet be more feedback controls of PI(4,5)P2 levels to be discovered."*

Technical standards: The work is done to a high technical standard.

© 2023. Published by The Company of Biologists under the terms of the Creative Commons Attribution License (https://creativecommons.org/licenses/by/4.0/).

#2.3: Does catalytically dead isoform of PIP4K2B and 2C also yield the same result as a catalytically dead version of PIP4K2A in Fig 1B?

In a word: yes. We have added these experiments, which are now presented in **Fig. 2A:**

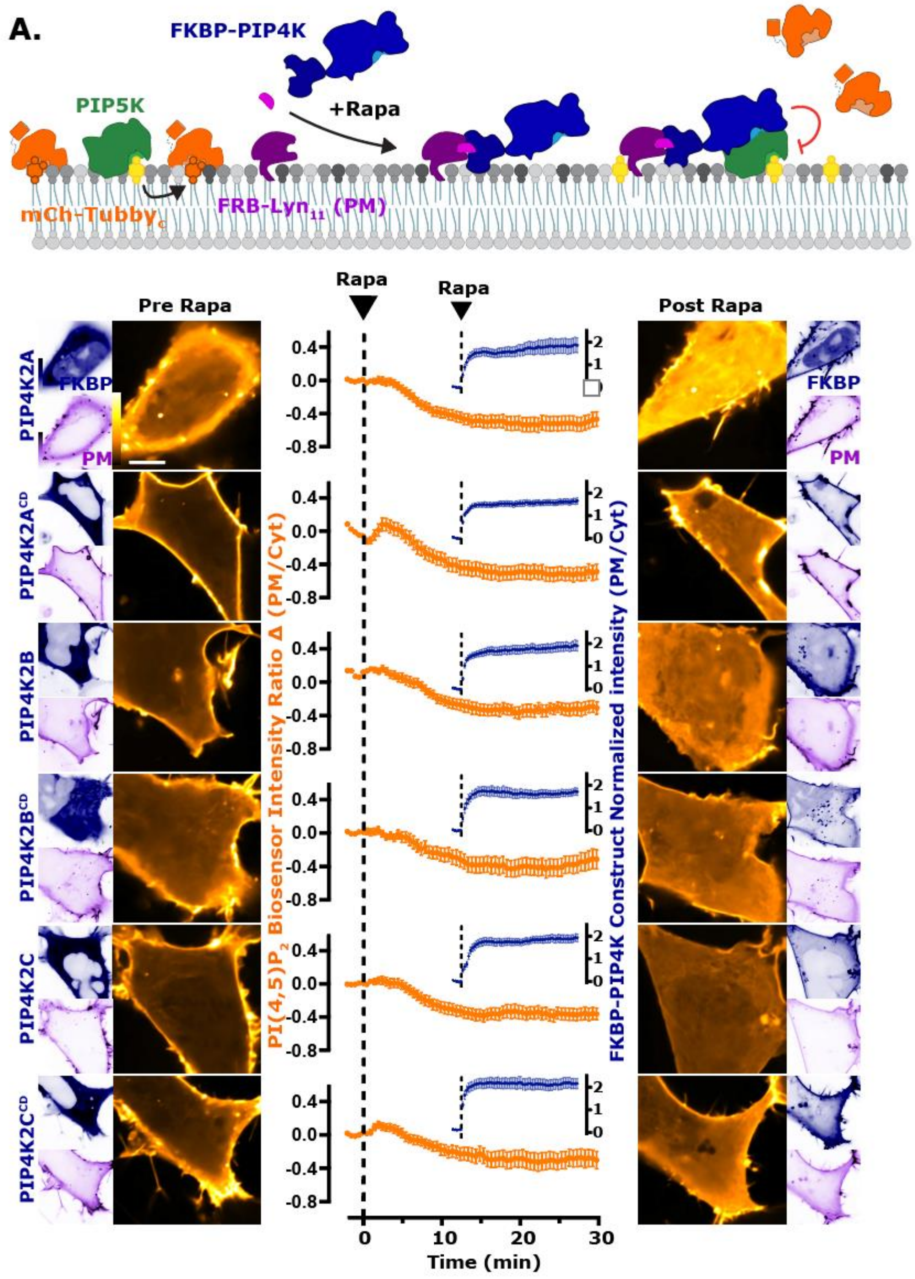

(**A**) PIP4K recruitment acutely inhibits PM PI(4,5)P2 levels. Cartoon schematics show the chemically induced dimerization (CID) system for FKBP- tagged PIP4K isoforms (A, B, C), which dimerize with the PM-anchored FRB- Lyn11 upon the addition of rapamycin (rapa). HEK293A cells were transfected with FKBP-tagged proteins, the high affinity PI(4,5)P2 indicator TubbyC and FRB-Lyn11, scale bar is 10 μm. During time-lapse confocal microscopy, cells were stimulated with 1 μM rapa as indicated. Graphs represent mean change in PI(4,5)P2 sensor intensity ratio (PM/Cyt) ± s.e. for 35-60 cells imaged across three independent experiments (orange). Inset graphs show PM recruitment of the FKBP-PIP4K (blue).

The results are described in the results in the **2nd paragraph of p. 4**: *"To directly test for*

© 2023. Published by The Company of Biologists under the terms of the Creative Commons Attribution License (https://creativecommons.org/licenses/by/4.0/).

*negative regulation of PIP5K activity by PIP4K in cells, we wanted to assay PI(4,5)P2 levels after acute membrane recruitment of normally cytosolic PIP4K paralogs. To this end, we triggered rapid PM recruitment of cytosolic, FKBP-tagged PIP4K by chemically induced dimerization (CID) with a membrane targeted FRB domain, using rapamycin [27]. As shown in **fig. 2A**, all three paralogs of PIP4K induce a steady decline in PM PI(4,5)P2 levels within minutes of PM recruitment. Catalytically inactive mutants of all three paralogs produce identical responses (**fig. 2A**)."*

***#2.4**: The labelling on y-axis for PI(4,5)P2 biosensor intensity ratio is PM/cell at some places, PM/Cyt or PM/Cyto in some places. It is recommended to make it uniform across all the panels.*

PM/Cyto was a typo, now corrected to PM/Cyt. PM/Cell and PM/Cyt are two subtly different metrics used to normalize PM fluorescence intensity across varying transient expression levels. This is clarified in the methods in the **3rd paragraph on p.22:** *"For confocal images, the ratio of fluorescence intensity between specific compartments was analyzed as described previously [59]. In brief, a custom macro was used to generate a compartment of interest specific binary mask through à trous wavelet decomposition[68]. This mask was applied to measure the fluorescence intensity within the given compartment while normalizing to the mean pixel intensity in the ROI. ROI corresponded to the whole cell (denoted PM/Cell ratio) or a region of cytosol (PM/Cyt), as indicated on the y axis of individual figures."*

***#2.5**: The claim that PI(4,5)P2 production is sufficient to recruit PIP4K2C to the PM can be ascertained further if one is able to do an experiment where PI(4,5)P2 is ectopically expressed in some compartment of the cell which is non-native to PI(4,5)P2 and as a consequence of this PIP4K2C is recruited to this non-native compartment.*

We have now removed the assertion that PI(4,5)P2 is sufficient to localize PIP4Ks to the membrane, since our conclusion is that the coincident presence of PI(4,5)P2 and PIP5Ks in the PM is what ultimately localizes the PIP4Ks. We did not detect recruitment of endogenous PIP4Ks to lysosomes when ectopic PI(4,5)P2 synthesis was induced, although fluorescence levels are so low as to be inconclusive, and therefore not appropriate for inclusion in the manuscript.

***#2.6**: In the entire figure 2, to establish that PI(4,5)P2 is necessary and sufficient for PM localisation of PIP4K, PIP4K2C is used as the PIP4K isoform on the basis that it is highly abundant in HEK293 cells. But PIP4K2A is localised mainly at the plasma membrane and here we are discussing about PI(4,5)P2 regulation at the PM . Can experiments be done with isoforms 2A and 2B as well? Can acute depletion of PI(4,5)P2 lead to the membrane dissociation of the isoform 2A as well? This will help us in understanding if there is an isoform specific difference in sensing PI(4,5)P2 levels which will help us in targeting specific isoform as therapeutic targets.*

We have now generated endogenously tagged PIP4K2A and PIP4K2B; these cell lines are characterized in the revised **fig. 3**:

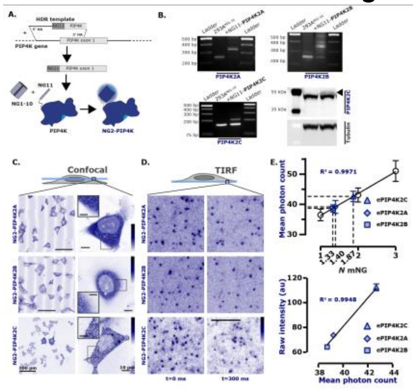

© 2023. Published by The Company of Biologists under the terms of the Creative Commons Attribution License (https://creativecommons.org/licenses/by/4.0/).

Fig. 3. Endogenously tagged PIP4Ks are associated with the PM. (**A**) Endogenous tagging of PIP4K. Brief cartoon schematic showing the mechanism of endogenous tagging employed for PIP4K with NeonGreen2 (NG2). The resulting cell lines were termed NG2-PIP4K. This strategy was used for all three isoforms of PIP4K. (**B**) For PIP4K2A and PIP4K2B isoforms, cells were genotyped with isoform specific forward and reverse primer yielding an edited product of ~350bp. For PIP4K2C, cells were genotyped with a NG2 specific forward primer and a PIP4K2C isoform specific reverse primer yielding an edited product of ~200bp. NG2-PIP4K2C cells were also probed with a PIP4K2C specific antibody showing the expected ~3 kDa shift in weight (arrowhead). (**C**) Confocal based characterization of NG2-PIP4K. Confocal images display the NG2-PIP4K (blue) in cells localized mainly to the cytosol, scale bar is 100 µm or 10 µm, as noted. In the case of NG2-PIP4K2C, slight association of the enzyme to the PM can be seen in the zoomed image, scale bar for zoomed images is 5 µm. (**D**) TIRF based characterization of NG2-PIP4K. When imaged live by TIRF, dynamic, diffraction limited spots are observed on the membrane (compare differential localization at 0 and + 300 ms), scale bar is 2.5 µm. (**E**) Endogenous PIP4Ks exists as heterogeneous populations. All three PIP4K isoforms have an intensity consistent with a mixed population of 1 or 2 mNG molecules when calibrated against single, dimeric or trimeric mNG molecules fused to a PI(4,5)P_2 binding domain. This correlates to the mean photon count of heterogeneously tagged cell populations (one or two alleles tagged with NG2[11]). Data are grand mean photon counts ± 95% C.I. for data acquired from 22-43 cells. Linear regression and resulting R^2 against grand mean values is also plotted. (**F**) Endogenous expression levels vary between PIP4K isoforms. The raw fluorescence intensity of NG2-PIP4K2A, NG2-PIP4K2B, or NG2-PIP4K2C in each tagged cell line was measured and plotted against the mean photon counts from E. Plotted pointes show the raw fluorescence intensity of each NG2-PIP4K isoform from 90 cells imaged across three independent experiments (circles display the mean and error bars representing s.e.). There is a positive correlation between the expression level of each isoform and its likelihood to be visualized as a monomeric vs a dimeric protein (Linear regression and resulting R^2 against mean values is plotted). PIP4K2A and PIP4K2B may exist as either homodimers or more likely heterodimers; whereas PIP4K2C more likely homodimerizes.

With the dependence on PI(4,5)P_2 for PM binding for all isoforms shown in **fig. 4**:

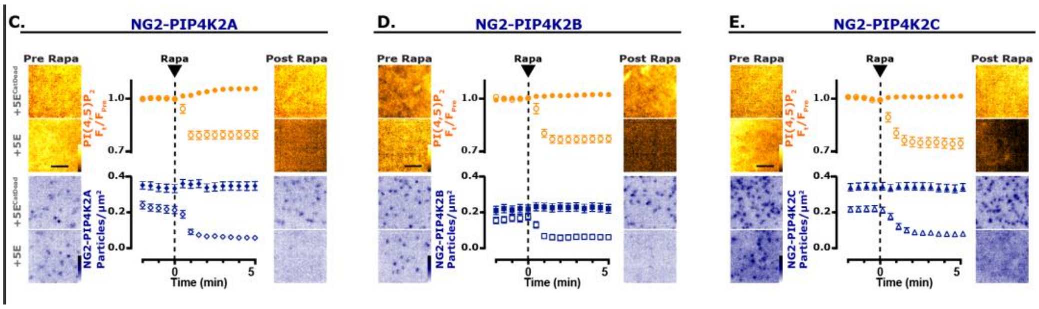

(**C**) Depletion of PI(4,5)P_2 causes NG2-PIP4K2A to dissociate from the membrane. NG2-PIP4K2A (blue) cells were transfected with FKBP-tagged proteins, the high affinity PI(4,5)P_2 indicator Tubby$_C$ (orange) and Lyn$_{11}$-FRB, scale bar is 2.5 µm. During time- lapse TIRF microscopy, cells were stimulated with 1 µM rapa, as indicated. Tubby$_C$ traces represent mean change in fluorescence intensity (F_t/F_{pre}) ± s.e. The NG2-PIP4K2A traces represent the mean change in puncta per µm^2 ± s.e. of 29-32 cells that were imaged across three independent experiments. (**D**) Depletion of PI(4,5)P_2 causes NG2-PIP4K2B to dissociate from the membrane. As in C, NG2-PIP4K2B (blue) cells were transfected with FKBP-tagged proteins, Tubby$_C$ (orange) and Lyn$_{11}$-FRB, scale bar is 2.5 µm; cells were stimulated with 1µM rapa, as indicated. Tubby$_C$ traces represent mean change in fluorescence intensity (F_t/F_{pre}) ± s.e. The NG2-PIP4K2B traces represent the mean change in puncta per µm^2 ± s.e. of > 32 cells that were imaged across three independent experiments. (**E**) Depletion of PI(4,5)P_2 causes NG2-PIP4K2C to dissociate from the membrane. As in C, NG2-PIP4K2C (blue) cells were transfected with FKBP-tagged proteins, Tubby$_C$ (orange) and Lyn$_{11}$-FRB, scale bar is 2.5 µm; cells were stimulated with 1µM rapa, as indicated. Tubby$_C$ traces represent mean change in fluorescence intensity (F_t/F_{pre}) ± s.e. The NG2-PIP4K2C traces represent the mean change in puncta per µm^2 ± s.e. of > 38 cells that were imaged across three independent experiments.

© 2023. Published by The Company of Biologists under the terms of the Creative Commons Attribution License (https://creativecommons.org/licenses/by/4.0/).

And increased binding by elevated PI(4,5)P2 levels shown in **fig. 5B**:

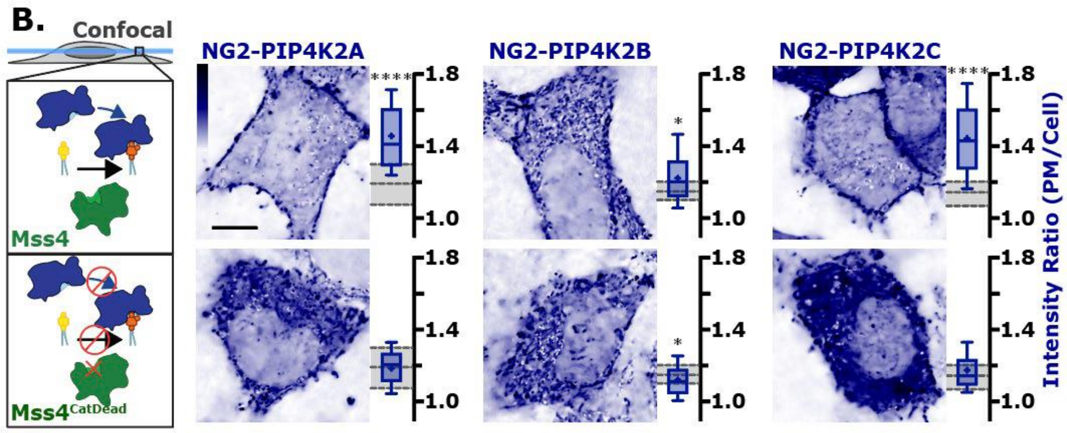

(**B**) Chronic enrichment of PI(4,5)P2 causes NG2-PIP4K isoforms to associate with the membrane. Cartoons show the expression of catalytically active or dead Mss4. Images show equatorial confocal sections of representative NG2-PIP4K cells transfected with Mss4, scale bar is 10 µm. Box and whisker plots show the mean fluorescence intensity ratio (PM/Cell) of the indictated NG2-PIP4K isoform from 90 cells images across at least three independent experiments (boxes display median and interquartile range, whiskers represent 10-90% of data and "+" represents mean; *P < 0.05; **P < 0.01; ***P < 0.001; ****P < 0.0001).

The results are described in the accompanying results text *"PIP4K are low affinity sensors of PM PI(4,5)P2"*, **pp.4-7**. In short, endogenous PIP4K isoforms behave similarly with respect to PI(4,5)P2-dependent PM recruitment.

#2.7: *In Figure 1A, it is shown that overexpression of a catalytically dead PIP5K 1A/1B/1C is still able to increase PI(4,5)P2 levels. In the figure 2E, expression of homodimeric mutant of PIP5K domain which is a way to increase catalytic activity of PIP5K, increases PI(4,5)P2 levels which is consistent with the inferences from Fig, 1 , but what is surprising is a catalytically dead variant not being able to do so. Why is there a discrepancy between Fig. 1A and Fig. 2E? If the homodimeric mutant is the reason, then it is not clear in the explanation.*

We have added the following clarification to the results on the second paragraph of **p.6:** "*We next tested for rapid binding to acutely increasing PI(4,5)P2 levels in living cells, using CID of a homodimeric mutant PIP5K domain (PIP5K-HD), which can only dimerize with itself and not endogenous PIP5K paralogs [34]. This domain also lacks two basic residues that are crucial for membrane binding [35], and only elevates PM PI(4,5)P2 when it retains catalytic activity (**fig. 5D**), unlike the full-length protein (**fig. 1A**).*" We currently do not fully understand why these well characterized residues of PIP5Ks are necessary for PM binding and inhibition by PIP4K. This is a focus of ongoing studies in the lab for the structural basis of PIP5K inhibition by PIP4K.

#2.8: *Show the loading control in Fig 2A western.*

We have added the loading control using alpha tubulin in the revised **fig. 3B**.

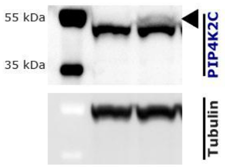

© 2023. Published by The Company of Biologists under the terms of the Creative Commons Attribution License (https://creativecommons.org/licenses/by/4.0/).

***#2.9**: In the figure 2D, in the legend OCRL is written. So, the labelling in the panel should also be changed to OCRL from INPP5E. It is intermixed.*

Reviewer 1 also spotted this inconsistency (#1.7): Purified OCRL was indeed in the supported lipid bilayer experiments. The figure (now **Fig. 4A**) and legend have been corrected – thank you for spotting the error.

***#2.10**: In the figure 2E, can the labelling be changed from HD to something more self-explanatory for homodimeric mutant of PIP5K domain?*

We prefer to keep the "HD" notation in the revised **figure 5D** for brevity's sake, but now define the abbreviation in the text in the **second paragraph of p.6:** "*…a homodimeric mutant PIP5K domain (PIP5K-HD)…*".

***#2.11**: In Fig. 2E, PIP5K expression is acute and in Fig. 2F Mss4 expression is chronic, both of which is able to recruit PIP4K2C to the plasma membrane. How can a likewise argument be drawn out of these two experiments when one is acute and the other one is a chronic expression? It is suggested to do an FRB-FKBP experiment for Mss4 as well.*

We agree with the reviewer that an FKBP-Mss4 would have been an excellent experiment. As can be seen from **Fig.** 1A, Mss4 is constitutively PM localized in mammalian cells. However, we were unable to identify a truncation of Mss4 that lost constitutive membrane binding whilst retaining catalytic activity. Therefore, we could only perform chronic overexpression as shown in **fig. 5B**. The lack of an acute demonstration is why we went on to develop the PIP5K-HD constructs, results of which are reported in **fig. 5D.**

***#2.12**: In the text, Fig. 2G and 2H is written for PIP4K2C, but in the corresponding panels and legends, it is an assay for purified PIP4K2A on SLBs. Kindly resolve the discrepancy.*

We thank the reviewer for spotting this discrepancy. PIP4K2A is the protein that was used in the SLB experiments now reported in **fig. 5A & C** and the accompanying results on **pp.5-6**. This is now corrected in the manuscript.

***#2.13**: Kindly explain a bit in detail why the baits were now targeted to ER-PM contact sites. It is not self-explanatory.*

We have now added a more detailed description to the **third paragraph of p. 7**: "*We therefore sought to distinguish between a direct PIP5K-PIP4K binding interaction versus PI(4,5)P2- induced co-enrichment on the PM. To this end, we devised an experiment whereby a bait protein (either PIP5K or control proteins) could be acutely localized to subdomains of the PM, with the same PI(4,5)P2 concentration. This was achieved using CID of baits with an endoplasmic reticulum (ER) tethered protein, causing restricted localization of the bait protein to ER-PM contact sites – a subdomain of the PM (**fig. 7B**).*"

***#2.14**: The conclusions for Fig. 3 most likely hints towards the possibility of PIP4K and PIP5K interaction being independent of PI(4,5)P2 levels. Well, Fig. 3C and 3D does suggest a direct interaction, but can other protein-protein interaction assays be used to establish the direct interaction of PIP4K with PIP5K such as FRET or Yeast two hybrid as assays scoring for interaction?*

We respectfully diverge from the reviewer's assessment of the data, presented in the revised **fig. 7**. **Figs. 7A & B** show PIP4K and PIP5K interacting in the context of a PI(4,5)P2 replete PM; **fig. 7C** shows this in the context of a PI(4,5)P2 replete SLB. Therefore, we make no assertion that the PIP4K/PIP5K is independent of PI(4,5)P2 levels. We also contend that the latter experiment is a more direct demonstration than a Y2H assay, or even FRET (which can occur among non-interacting proteins localized to a membrane surface, see e.g. 10.1074/jbc.m007194200).

© 2023. Published by The Company of Biologists under the terms of the Creative Commons Attribution License (https://creativecommons.org/licenses/by/4.0/).

***#2.15**: Conceptually a direct interaction can be explained to some extent from Fig. 3 but extending it to be an inhibitory interaction is not right without a direct experiment. Can an experiment be done with PI4P enriched SLB, wherein you put just PIP5K purified protein vs PIP5K+PIP4K combination and measure the % mol of PI(4,5)P2 produced using a probe. That will be suggestive of a negative interaction.*

This is a great experiment, the results of which are reported in **fig. 2C**, described in the **third full paragraph of p. 4**: *"To more directly examine inhibition of PIP5K by PIP4K, we tested activity of purified PIP5K1A on PI4P-containing supported lipid bilayers (SLBs). Addition of PIP4K2A exhibited delayed inhibition of PIP5K1A activity (**fig. 2C**): Once PI(4,5)P$_2$ reached approximately 28,000 lipids/µm^2 (~2 mol %), PIP5K dependent lipid phosphorylation slowed down, which doubled the reaction completion time (**fig. 2C**, right). In contrast, we observed no PIP4K dependent inhibition of Mss4 (**fig. 2C**, inset). These data recapitulate the prior finding that PIP4K only inhibited purified PIP5K in the presence of bilayer-presented substrate [25]. We therefore hypothesized that inhibition of PIP5K by PIP4K requires recruitment of the latter enzyme to the PM by PI(4,5)P$_2$ itself."*

***#2.15**: In Figure 3B, the FRB tagged constructs are magenta coded and PIP4K2C is cyan. Kindly change the labelling of the FRB constructs on the y axis to magenta so that it goes with what is written in the legend. It will also be appreciated to show a colocalization quantification between the magenta (FRB constructs) and cyan (PIP4K2C) post rapamycin addition and not just the intensity for ER-PM recruited PIP4K2C.*

These modifications and some additional points have been added in response to reviewer 1's **#1.4** to the revised **fig. 7B**. Note, we quantified the co-localization with an ER-PM contact site marker, MAPPER. Co-localization with the FRB-tagged construct would be misleading, because this construct is localized across the membrane at the start of the experiment and would thus have a high degree of co-localization. As can be seen from the inset graphs in the new analysis, however, all FRB-tagged constructs co-localize with MAPPER after rapamycin addition, but only FRB-PIP5K1A causes endogenous PIP4K2C to increase co-localization with this compartment.

***# 2.16**: Again, in the text , the description is written for PIP4K2C but in the result panel and legend (Fig. 3C and Fig. 3D), PIP4K2A is mentioned. Kindly resolve the discrepancy*

We have corrected the results text on the last paragraph of **p. 7**: *"Finally, we also demonstrate that PIP4K2A binding to PI(4,5)P$_2$-containing supported lipid bilayers was greatly enhanced by addition of PIP5K to the membranes (**fig. 7C**), but not by Mss4 (**fig. 7D**)."*

***# 2.17**: In the Fig. 4B, it will be appreciated to show statistical significance in terms of R2 value for commenting on the linear response.*

"Linear response" was not the best description of what we were trying to articulate in the revised **fig. 8B**; we have now amended the results in the **2nd paragraph of p.8** to read: *"Of these, Tubby$_C$ showed the largest degree of change in PM localization across all changes in PI(4,5)P$_2$ levels (**fig. 8B**)."*

***#2.18**: Discussion can be in general a bit more detailed which is suggestive of future experiments to do that can shed more light on the interaction such as which residues in PIP4K interacts with PIP5K to negatively regulate it.*

The revised manuscript contains a greatly expanded discussion, as described in detail in our responses to comments **#1.2b**, **#2.1** and **#2.2.**

***#2.19**: In the discussion, more light can be shed on the fact that Mss4 in spite of being a 5- kinase is not negatively regulated by PIP4K and the fact that PIP4K is present only in metazoans suggests that this fine tuning of PI(4,5)P2 levels is specific to*

© 2023. Published by The Company of Biologists under the terms of the Creative Commons Attribution License (https://creativecommons.org/licenses/by/4.0/).

metazoans. Another insight could be in the direction, that Fig 4. tells PI3K, but not calcium signaling is modulated by this fine tuning and interestingly class I PI3K is also an enzyme specific to metazoans. Hence, unlike yeast, metazoans rely on growth factor signalling processes, hence regulation of PI(4,5)P2 by PIP4K and hence Class I PI3K and PI(3,4,5)P3 could be a process relevant to metazoans.

We have addressed the restriction of PIP4K to holozoa as described in our response to ***#2.2***, wherein we describe a previously proposed paralogous mechanism in fungi. The reviewer's point about the homeostatic process being related to class I PI3K signaling in growth control of multicellular organisms is interesting, but the presence of the PIP4Ks in some unicellular organisms complicates this view. We are of the view that a discussion of this important topic is a little nuanced for inclusion in the current manuscript.

Reviewer #3

Summary: *Using state of the art imaging techniques the authors try to address how cells sense PI(4,5)P2 levels and regulate PIP5Ks to maintain an optimal level since any dysregulation of PI(4,5)P2 levels can have significant effects on the functioning of the cell and led to numerous disease states, such as cancers.*

The key conclusions are convincing and importantly validate previous disputed findings made by Wang et al. (Cell Reports 2019) using different and more rigorous methods, however unfortunately due to the Wang et al publication the overall novelty of this study is lacking. A suggestion to the authors is to state/explain with text more clearly how their findings are more precise and higher quality than the previous report and why their findings are necessary and significant to drive the field forward.

We have revised the manuscript to more clearly state our novel finding that PIP4Ks are PI(4,5)P2 sensing proteins that inhibit PIP5Ks on the membrane in a PI(4,5)P2-dependent manner, which was not previously described in the literature.

Further, experiments in the study were performed in vitro in cultured cells using overexpression methods making the physiological significance a bit unclear and the enthusiasm of the main discovery dampened. With that being said these findings are worthy of publication in order to advance the field and understanding of how the PIP kinase families are regulated and maintain PIP2 homeostasis which is important for life.

We feel that this assessment is slightly unfair, since most of the key experiments have been validated using purified proteins in supported lipid bilayers, and endogenous proteins were studied using genomic tagging approaches, rather than over-expression.

Minor and easily addressable experiments should be performed by the authors the following. Further, many of these experimental issues can easily go in supplemental materials

#3.1: *Include western blots for the constructs to compare expression levels.*

We agree that it is important to take into account differences in expression levels for the experiments presented in **fig. 1**. However, since these are single cell assays, Western blotting of whole populations of transiently transfected cells is not the best control. Instead, having acquired the images under consistent excitation and detection parameters, we compared the fluorescence intensity, expressed as relative expression in **Fig. 1A** and **C**, which is discussed in the results text in the **first two paragraphs of the results on p. 3**: *"Notably, expression of the catalytically inactive mutants was usually somewhat less strong compared to the wild-type enzymes, yet effects on PI(4,5)P2 levels were similar (**fig. 1A**)."* and *"Again, differences in expression level between isoforms do not explain differences in activity, since all achieved comparable expression levels as assessed by fluorescence intensity (**fig. 1C**)."*

© 2023. Published by The Company of Biologists under the terms of the Creative Commons Attribution License (https://creativecommons.org/licenses/by/4.0/).

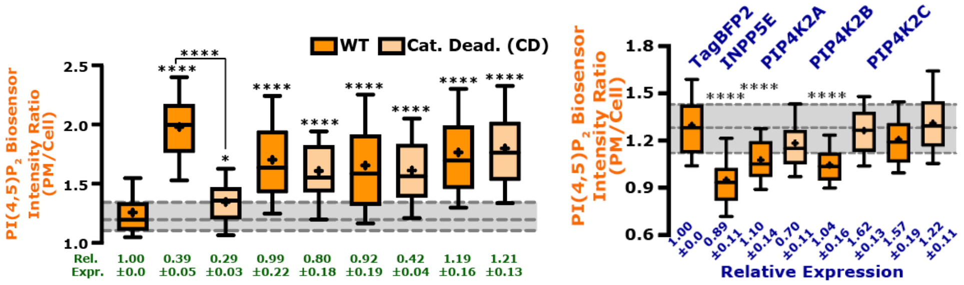

#3.2:* *For Figure 1A, what is the source of the observed increase in PI(4,5)P2, how do the authors take into account the role of endogenous PIP5Ks?

We added a new experiment in the revised **Fig. 1B** showing that the increased PI(4,5)P2 occurs at the expense of PM PI4P:

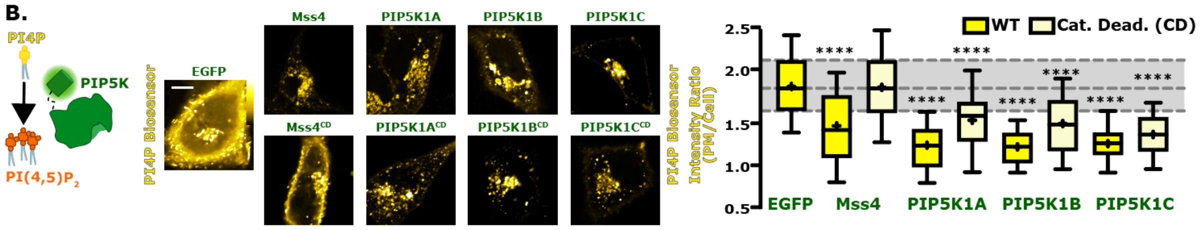

(**B**) PIP5Ks reciprocally decrease PM PI4P independently of catalytic activity. Cartoon denotes the catalytic activity of PIP5K. Images show equatorial confocal sections of HeLa cells expressing the high affinity P4Mx2 PI4P sensor (yellow), co-transfected with EGFP-tagged catalytically active or dead PIP5K constructs (yeast Mss4 or mammalian A, B, or C isoforms), or EGFP alone as control, scale bar is 10 µm. Decreased PI4P is apparent from loss of P4Mx2 fluorescence at the PM. Box and whiskers from 88-90 cells imaged across at least three independent experiments as in A.

This is described in the **first paragraph of the results on p.3**: *"PI(4,5)P2 levels are expected to increase at the expense of PM PI4P levels when over-expressing any of the three isoforms of human PIP5K (A-C) or the single paralog from the budding yeast, Saccharomyces cerevisiae (Mss4). Indeed, this was precisely what we observed (**fig. 1A and B**, statistics reported in **tables 1 and 2**)."*

The role for endogenous PIP5Ks is clarified on the sentence that spans **pp. 3-4**: *"We therefore reasoned that saturation of endogenous, inhibitory PIP4K molecules by PIP5K over-expression, regardless of catalytic activity of the PIP5K, would free endogenous, active PIP5K enzyme from negative regulation (**fig. 1D**)."*

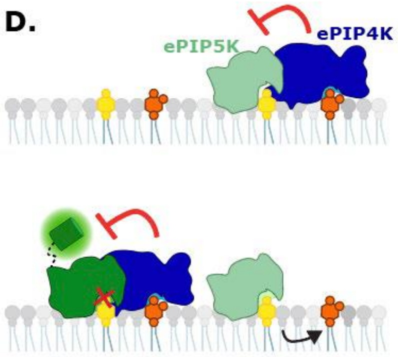

#3.3:* *For Figure 1B, could the authors comment on the intracellular distribution of PI(4,5)P2. How are they able to reliably distinguish their signal between plasma membrane and intracellular localizations and conclude that PIP2 on the plasma membrane is decreased?

© 2023. Published by The Company of Biologists under the terms of the Creative Commons Attribution License (https://creativecommons.org/licenses/by/4.0/).

As detailed in the now expanded methods section covering image analysis on **p. 22**, our analysis specifically quantifies fluorescence in the plasma membrane.

#3.4: Please include statistics for all image- based quantitation analysis.

We have added details of statistical analysis and tabulated the results, as detailed in our response to **#1.1.**

#3.5: Could the authors comment on the ability of PIP4K to have affinity for its own product? How does PIP4K sense membrane PI(4,5)P2 since these kinases are mostly cytoplasmic?

We have added a comment to the **1st paragraph of the Discussion on p.9**: *"PIP4K's low affinity and highly co-operative binding to PI(4,5)P2 makes it an excellent sensor for tonic PI(4,5)P2 levels. It is poised to sense PI(4,5)P2 generated in excess of the needs of the lipids' legion effector proteins, ensuring these needs are met but not exceeded. Nevertheless, the relatively low PIP4K copy number of around 2.5 x 10^5 per cell [29] is a small fraction of the total PI(4,5)P2 pool, estimated to be ~10^7 [33], ensuring little impact on the capacity of the lipid to interact with its effectors."*

#3.6: Do the authors have any other experiments to substantiate the binding of the two PIP kinases, similar to the Wang et al findings? Is the N-term motif required? Is it possible to disrupt that interaction and show the phenotype?

We do not have additional, conclusive experiments to share at this time, and believe that characterization of the inhibitory interaction is beyond the scope of the current manuscript. We do however add a comment on this topic to the **1st paragraph of p. 11**: *"Regardless, a full understanding of paralog selectivity will need to be driven by a detailed structural analysis of the interaction between PIP4Ks and PIP5Ks - which is not immediately apparent from their known crystal structures, especially since PIP4Ks and PIP5Ks employ separate and distinct dimerization interfaces [50]."*

#3.7: With the overexpression studies in Figure 1, do the authors see any changes in signaling when they just overexpress PIP5Ks versus in combination with PIP4Ks to show that the changes in plasma membrane PI(4,5)P2 can affect downstream signaling?

We agree with the reviewer that attenuating PIP5K-mediated PI(4,5)P2 increases with PIP4K should affect downstream signaling. However, we believe that these will not add additional insight compared to the already included experiments (**fig. 8**), whereby signaling output in response to graded changes in PI(4,5)P2 levels was investigated.

Reviewer #3 (Significance (Required)): *Overall, as mentioned above because of the 2019 Wang et al report the novelty is diminished, however using completely alternate methods and sophisticated microscopy this body of work indeed advances the field and provides further believable evidence of the PIP kinase families communicating in higher organisms which is required to maintain PIP2 levels shedding light on many of the findings that were previously unexplained surrounding the PIP4K studies. Further, the use of biosensors to describe these findings are new and will enable others in the field to begin to use such tools to investigate potential crosstalk between other lipid kinases.*

As we argued in the general comments, we do feel that this evaluation misses the key finding that PIP4Ks are PI(4,5)P2 sensors, and that this regulates PIP5K regulation as part of a feedback loop.

© 2023. Published by The Company of Biologists under the terms of the Creative Commons Attribution License (https://creativecommons.org/licenses/by/4.0/).

First decision letter

MS ID#: JOCES/2023/261494
MS TITLE: A Novel Homeostatic Mechanism Tunes PI(4,5)P2-dependent Signaling at the Plasma Membrane
AUTHORS: Rachel C Wills, Colleen P Doyle, James P Zewe, Jonathan Pacheco, Scott D Hansen, and Gerald R Hammond
ARTICLE TYPE: Research Article

Thank you for sending your manuscript to Journal of Cell Science through Review Commons.

Thank you for sending your revised Review Commons manuscript to JCS. I think that your revisions have fully addressed the reviewers concerns and there is no need to go back to the reviewers. I am therefore happy to tell you that your manuscript has been accepted for publication in Journal of Cell Science, pending standard ethics checks.

© 2023. Published by The Company of Biologists under the terms of the Creative Commons Attribution License (https://creativecommons.org/licenses/by/4.0/).
